# Melissopalynology and antioxidant properties used to differentiate *Schefflera abyssinica* and polyfloral honey

**DOI:** 10.1371/journal.pone.0240868

**Published:** 2020-10-28

**Authors:** Demelash Hailu, Abera Belay

**Affiliations:** Department of Food Science and Applied Nutrition, Addis Ababa Science and Technology University, Addis Ababa, Ethiopia; Beijing Foreign Studies University, CHINA

## Abstract

Honey can be categorized as monofloral and polyfloral honey. There is a strong interest in science and commerce, to further differentiate honey. In the present study, *Schefflera abyssinica* and polyfloral honey from Sheka Forest, Ethiopia was investigated. Botanical origin was determined based on Melissopalynology. Refractive index, moisture, sugars, ash, pH, free acidity, hydroxymethylfurfural, optical density, diastase activity, protein, and color were determined based on the standard method of the international honey commission (IHC) and AOAC. Antioxidant activity and Antioxidant content were determined using UV- visible spectroscopy. The level of pollen dominancy for monofloral honey (*Schefflera abyssinica*) ranged from 76.2 to 85.8%. The polyfloral honey stuffed with a variety of pollen grain ranged from 2.2% (*Coffea arabica*) to 23.2% (*Schefflera abyssinica*). *Schefflera abyssinica* honey contained more total phenolic compounds (75.08 ± 2.40 mg GAE/100g), and total flavonoids (42.03 ± 1.49 mg QE/100 g), as well as had stronger DPPH (44.43 ± 0.97%) and hydrogen peroxide (78.00 ± 4.82%) scavenging activity. The principal component analysis revealed that *Schefflera abyssinica* honey associated with the antioxidant properties of total phenolic, total flavonoids, DPPH, and H_2_O_2.,_ which revealed that floral honey sources can essentially differentiated by antioxidant patterns. The higher electrical conductivity (0.42 ± 0.02 mS/cm), ash (0.41 ± 0.05 g/100g), pH (4.01 ± 0.08), optical density (0.26 ± 0.03) and diastase activity (5.21 ± 0.17 Schade units) were recorded in polyfloral honey. *Schefflera abyssinica* and polyfloral honey satisfy the requirement of national and international standards. The pollen analysis in combination with antioxidant properties distinguishes *Schefflera abyssinica* from polyfloral honeys.

## Introduction

Honey is a natural product consumed without the addition of any ingredient, and is characterized by its complex composition, which varies according to the bee species, geographical region and available floral source [1, 2]. Ordinarily, honey categorized as monofloral (when the majority of the honey produced from single plant species) and polyfloral (honey produced from the contribution of different plants) honey [3, 4].

According to Kortesniemi et al. [5], botanical origin has an impact on the sensory, physicochemical, and bioactive properties of honey. The origin of honey is an important indicator of quality, authenticity, bioactive potential and commercial value. In addition, there is a conventional standard developed by CA, EU, and Ethiopian standards [6]. The current international standards demand the setup of quality control protocols based on palynological and physicochemical characteristics of honey [6, 7].

Honey is a source of natural antioxidants with application in human health, and in the prevention of deteriorative oxidative reactions [8]. Antioxidant properties are strongly related to the chemical composition, which in turn, depends on the floral source and environmental factors [9].

Monofloral and polyfloral honey differ in their chemical composition, which is accounted to plant source, season, and geographical origin. The main compositions of honey are sugars (mainly, fructose and glucose) and water. The minor chemical component, which actually determines its value or class, of honey is strongly dependent on the floral/botanical origin or nectarous plant [2, 7]. Monofloral honey usually regarded as a more valuable class, because it offers people to choose what flavor they prefer. These days, the merits of honey determined by their botanical origin [10]. When the honey has been designated according to floral source and geographical origin, it will have the quality, traceability, and acceptance by consumers [1, 6].

The Quality of honey is qualified using EU, CA, and Ethiopian standards. Bogdanov [11] has therefore proposed certain constituents as quality criteria for honey. These include: moisture content, water-insoluble solids, electrical conductivity, reducing sugars, sucrose content, free acid, proline content, hydroxymethylfurfural (HMF), and diastase activity [12–14]. In addition to these, there is keen interest to consider the botanical and geographical origin, color, phytochemicals and sensorial properties of honey as a quality marker [6]. Belay et al. [15] reported the physicochemical properties of Harenna forest honey, Bale, Ethiopia. However, the monofloral and polyfloral honey collected from Sheka forest is not investigated. To our knowledge, research or marker was not set to differentiate the monofloral *Schefflera abyssinica* and polyfloral honey. There is a deception in the honey industry, worldwide. Polyfloral honey presented/substituted as a monofloral honey. Accordingly, the identification of honey markers can be used to trace and differentiate honey. The purpose of this study was to investigate the quality and characteristics of honey, based on botanical origin, antioxidant and physicochemical properties, which is used to differentiate *Schefflera abyssinica* and polyfloral honey.

## Material and methods

### Honey sample

**Fig 1:**
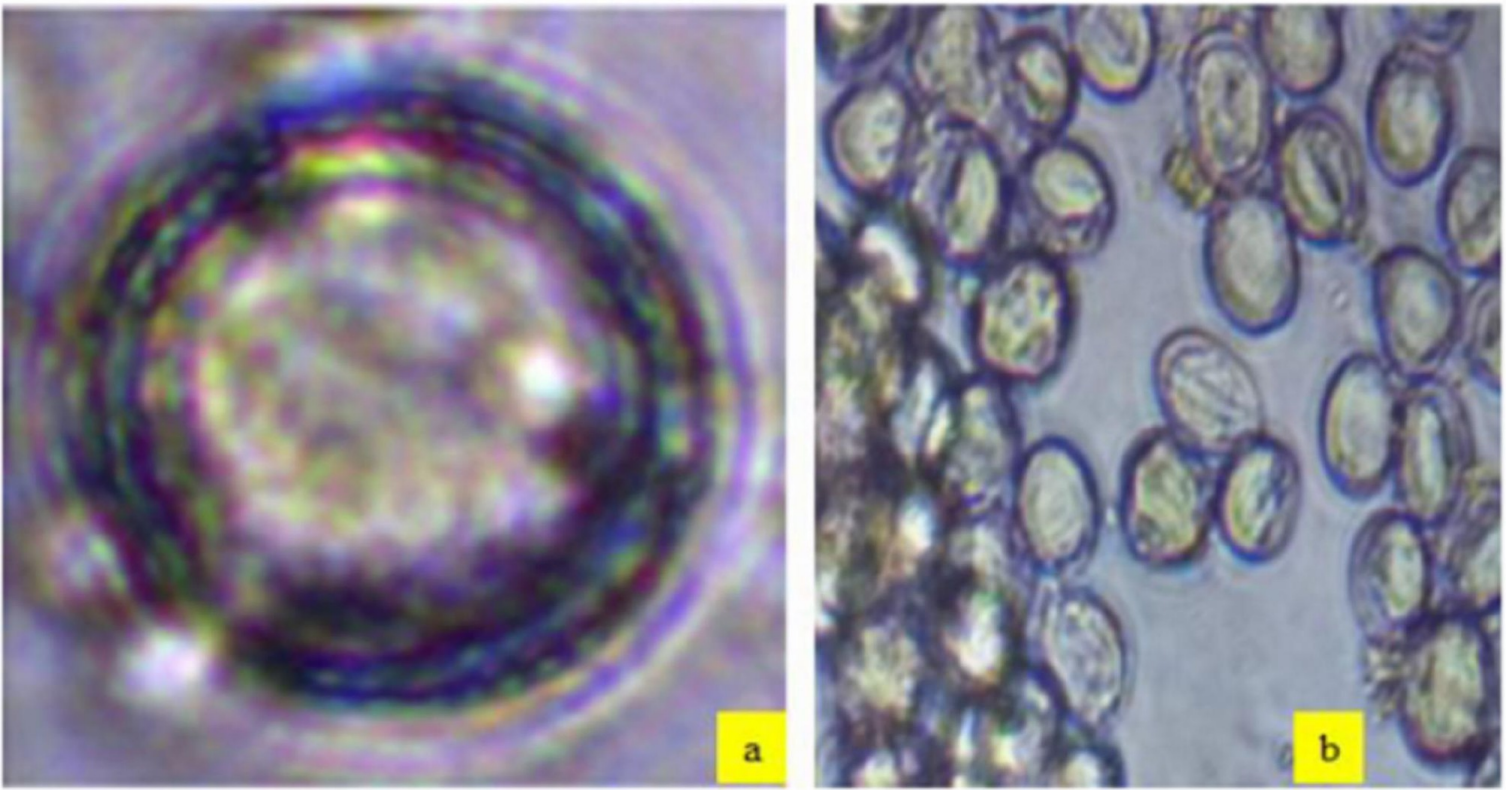
Pollen morphology of *Schefflera abyssinica* monofloral honey (a) and polyfloral honey (b).

Honey samples were collected on the collaboration of the Addis Ababa Science and Technology University, Department of Food Science and Applied Nutrition and Sheka Zone Livestock and Fishery Bureau. Accordingly, eighty honey samples (n = 80) were collected from Sheka forest, which is located in Sheka Zone, Ethiopia, and categorized based on botanical origin. From these honey samples, ten monofloral *Schefflera abyssinica* honey pollens (Fig 1A) were selected based on the pollen dominance level. In addition, another ten polyfloral honey pollens were also chosen based on the number of multifloral pollen (Fig 1B) represented in the honey samples. These honey samples stored at -20°C, until further analysis, to avoid the effect of laboratory changes on the chemical composition and physical properties of honey samples [16]. No specific permissions were required, for these locations/activities, and all honey harvesting and collection were performed without causing any harm to the honeybees and the forest. This field study did not endangered biodiversity. In Sheka, beekeeping has been delivering large benefits to the people and the biodiversity, for years.

### Sample analysis

#### Floral origin

Pollen analysis of honey carried out using Belay et al. [17]. Accordingly, ten gram of honey was weigh using a centrifuge tube and dissolved in 20 ml of warm distilled water (20–40°C). The solution was centrifuged at 2060 g (3500 rpm) for 10 minutes and the supernatant was decanted. Twenty ml water was added again to completely dissolve the remaining sugar crystals and centrifuged at 2060 g (3500 rpm) for 5 minutes and supernatant was removed. The sediment spread evenly using a sterile micro spatula on the microscope slide and the sample was dried for a while. Thereafter, one drop of glycerin jelly added to the coverslip, and the pollen grains were identified using pollen atlas [18]. The pollen count has done under a microscope (ZEISS, Germany). The percentage of pollen types in each honey sample calculated based on the total number of 500 pollen grains counted in each sample [17]. The dominant honey plant pollen of *Schefflera abyssinica* and polyfloral honey pollen presented in Fig 1A and 1B. Accordingly, ten honey samples (*Schefflera abyssinica*), which had 45% or more dominant pollen, and ten polyfloral honey were selected for laboratory analysis.

### Antioxidant content

#### Total phenolic content

The total phenolic content in honey was determined using Folin–Ciocalteu method in an alkaline environment [19]. About 100μL of honey extract (2.5 g of honey in 25 mL of water) was mixed with 50μL of Folin–Ciocalteu reagent (Concentration 2N) for 3 min. Then, 100μL of 35 g/100 mL sodium carbonate (Na_2_CO_3_) added, (final volume of 2.5 mL of water) and incubated at room temperature for 1 h. Gallic acid (0–100 μg/mL) was used as a standard to establish the calibration curve, and absorbance was measured at 765 nm against the blank using UV Spectrophotometer (Biochrom 80-7000-30, Cambridge England). The results expressed as mg gallic acid equivalent/100 g of honey.

#### Total flavonoids content

The total flavonoids in honey were determined using a modified photometric method [20]. About 150μL of 10% AlCl_3_.6H_2_O the solution in methanol was mixed with the 100μL honey extract (2.5 g of honey/25 mL of water). Then, 75mL of 5% NaNO_2_ solution for 5 min, afterward add 500μL 1 M NaOH in a final volume of 2 mL of water. Quercetin (0–100 μg/mL) was used as a standard to establish the calibration curve. Absorbance was measured at 510 nm using UV Spectrophotometer (Biochrom 80-7000-30, Cambridge England). The total content of flavonoids was expressed as mg quercetin equivalent (CE)/100 g honey.

### Antioxidant activity

#### 1, 1-diphenyl-2-picrylhydrazyl (DPPH) radical scavenging activities

1, 1-diphenyl-2-picrylhydrazyl (DPPH) radical scavenging activities were performed using Meda et al. [19]. Honey samples dissolved in water at concentrations from 20 to 120 μg/ml, and were mixed with 4 ml of 0.004% DPPH. Pure L-ascorbic acid standard was used as a reference. The mixtures were shaken vigorously and left for 30 min at room temperature in the dark, after which the absorbance of the remaining DPPH was measured at 517 nm against a blank using UV Spectrophotometer (Biochrom 80-7000-30, Cambridge England). The radical scavenging activities of DPPH radical, expressed as % inhibition, were calculated from the following equation.

$$\%\;\text{inhibition}\;=\frac{Abs\;blank-Abs\;sample}{Abs\;blanh}x\;100$$ (1)

where Abs blank = blank absorbance at 517 nm; Abs sample = sample absorbance at 517 nm.

#### Hydrogen peroxide scavenging activity

Hydrogen peroxide scavenging activity examined according to the method described by Ruch, et al. [21]. Accordingly, a solution of hydrogen peroxide (40 mM) was prepared in phosphate buffer saline (pH 7.4). A series of a various concentrated solution of each of the honey sample (1000ppm, 800ppm, 600ppm, 400ppm, 200ppm and 20ppm) were prepared in ethanol (95%) and added (1 ml) to the hydrogen peroxide solution (40 mM). The absorbance of hydrogen peroxide at 230 nm was determined after 10 minutes against a blank solution using UV Spectrophotometer (Biochrom 80-7000-30, Cambridge England). Ascorbic acid was used as standard, and the blank was prepared in phosphate buffer without H_2_O_2_. All the experiments carried out in duplicate.

The percentage of scavenged hydrogen peroxide was calculated by using the following equation.

$$\text{Percentage}\;\text{of}\;\text{scavenged}\;H_{2}O_{2}=\left[\frac{Ai-At}{Ai}\right]x\;100$$ (2)

Where Ai = absorbance of control; At = absorbance of test honey.

#### Inhibitory concentration

IC_50_ (Inhibitory concentration), is a measure of the potency of a substance in inhibiting a specific biochemical function by 50%, and computed based on the method stated by Al-Farsi et al [22]. IC_50_ was calculated by considering the dose-response curves obtained by plotting the percentage of inhibition versus concentration.

### Physicochemical properties

#### Moisture

The moisture content of honey samples was determined according to AOAC [23] method 969.38, using a Refractometry (KRUSS, Germany), thermostated at 20°C and regularly calibrated with distilled water. Honey samples were homogenized and placed on the surface of the prism of the refractometry. After 2 minutes, the refractive index (RI) for moisture was determined. The RI of distilled water (1.3330) used as a reference, after the measurement of four honey samples (eight measurements). The instrument checked with distilled water, and the value of the refractive index of the honey sample was determined using a standard table designed for this purpose, AOAC [23].

#### Sugar profile

Sugars profile were determined using high-performance liquid chromatography (HPLC- 1260 Infinity Series Agilent Technologies, Germany) equipped with a differential refractive index (DRI) detector [23]. Five-gram honey was taken from a properly homogenized sample and dissolved in Acetonitrile: water (70:30, v/v). The solution of each honey sample was filtered using a syringe filter (0.45 μm) and transferred to autosampler vials for HPLC determination of the sugars. The HPLC chromatogram peaks, identified by comparing the retention times obtained from standards, used to determine sugars.

#### Optical Density (OD)

The optical density of honey measured based on EI Sohaimy et al. [24]. Accordingly, 1 g of honey diluted in 9 ml of distilled water, and centrifuged for 10 min at 1510 g (3000 rpm). The absorbance of the filtrate supernatant measured at 530 nm against distilled water as a blank, using a spectrophotometer (Biochrom 80-7000-30, Cambridge England). The value calculated by subtracting the absorbance of the blank from the sample solution.

#### Electrical conductivity

Electrical conductivity was measured based on Bogdanov et al. [11]. Conductivity meter (AD 8000 pH /MV/EC/TDS & T^0^ Bench Meter, Romania) was used to determine the electrical conductivity of honey. Anhydrous honey (20 g) diluted in distilled water, and the solution transferred quantitatively to 100 ml volumetric flask and makeup to volume with distilled water. The conductivity cell was thereafter immersed in the sample solution and the conductance in mS read after temperature equilibrium had been reached [11]. Conductance was calculated in mS/cm as follows:

SH=KXG (3)

where SH = electrical conductivity of the honey solution in mS/cm, K = cell constant in cm^-1^ and G = conductance in mS.

#### pH and free acidity

pH of the aqueous honey solution (10g/75 ml) determined by using glass electrode after calibration with standard buffer solution pH 4, 7, and 10 (AOAC, 1990 method 962.19). Free acid (meq of acid/1000 g) was determined by dissolving honey sample (10 g/75 mL distilled water) and titrating with standardized 0.1 M NaOH to pH 8.3 using pH glass electrode attached to pH meter (AD 8000 pH /MV/EC/TDS & T^0^ Bench Meter, Romania) as endpoint indicator [23].

#### Hydroxymethylfurfural

Hydroxymethylfurfural (HMF) was determined using high performance liquid chromatography (HPLC- 1260 Infinity Series Agilent Technologies, Germany) based on international honey commission, Bogdanov [11] at the 285 nm using DAD (UV detector). Accordingly, 10 g of the honey sample was taken into a 50 ml beaker and dissolved the sample in 25 ml of water and transfer quantitatively to a 50 ml volumetric flask, and makeup using distilled water, and filter through a 0.45 μm membrane filter, and ready for chromatography. The HMF content of the sample calculated by comparing the corresponding peak areas of the sample and those of the standard solutions.

#### Ash

Ash Content of the honey samples was conducted based on AOAC [23] method 920.181. Accordingly, 5 g of honey sample was weighed (M_0_) and added into the dish. Then, water and other volatile components removed by preliminary carbonization using a hot plate at 350°C. After the preliminary ashing, the sample was ashed using a muffle furnace (Biobase DR 6300-T, Hamburg) at 600°C for 3 hrs. Dish with the ash was then cooled in a desiccator for 30 minutes and the weight was recorded (M_1_). Ash (% by mass) was calculated using the following formula:

$$\%\;Ash=\frac{M1-M2}{M0}\;x\;100$$ (4)

Where M_1_ = weight of the ash and crucible, M_2_ = weight of empty crucible, M_o_ = weight of the sample taken for the test.

#### Protein

The total protein content was measured using AOAC [23] method 962.18 based on the conversion of the organic nitrogen present in the sample to (NH_4_)2SO_4_. One gram of honey was taken and digests by H_2_SO_4_ (10 ml, 95–98%) with hydrogen peroxide and mixed catalyst and digest at 370°C for 3 hr. The resulting solution then distilled after adding NaOH (40%), and the distillate was collected in a flask with H_3_BO_3_ (5%) and mixed indicators. Finally, the mixture was titrated with H_2_SO_4_ (0.1 N). The percentage of nitrogen quantified was converted into protein content by multiplying with a conversion factor of 6.25.

#### Color

The color of honey was determined according to Karabagias et al. [25]. Accordingly, aqueous honey solutions (50%, w/v) were heated to 50°C to dissolve the sugar crystals and the samples were rapidly cooled to room temperature and the absorbance was read at 635 nm against water as a blank using UV Spectrophotometer (Biochrom 80-7000-30, Cambridge England). The absorbance was converted and classified according to the Pfund scale [26]. The conversion of the absorbance values (A_635_) was done using the following formula.

mmPfund=−38.70+371.39xAbs (5)

where Abs = absorbance of sample at 635 nm.

#### Total soluble solids (Brix)

Soluble solid (Brix) content of honey samples were determined according to the International Honey Commission, Bogdanov [11] Refractometry (KRUSS, Germany), thermostated at 20°C regularly calibrated with distilled water, was used to measure directly the °Brix.

#### Diastase activity

Diastase activity was performed by Phadebas, based on Harmonized method of international honey commission, Bogdanov [11] using the spectrophotometric method, in which an insoluble blue-dyed starch hydrolyzed by the enzyme; yielding blue water-soluble fragments. One gram of honey weighed into a 100 mL volumetric flask, dissolved in the acetate buffer solution and filled to the mark. Five ml of this solution was transferred to a test tube and placed in the water bath at 40°C. Acetate buffer solution prepared by dissolving 13.6 g of sodium acetate trihydrate in 1 L of distilled water and the pH was adjusted to 5.2 by glacial acetic acid (1–2 mL). A blank was prepared by placing 5 mL aliquot of the acetate buffer in another test tube, which is treated exactly like the sample solution. Phadebas tablets were added to both solutions using tweezers, and the timer started. Both solutions were stir in the reagent mixer until the tablet disintegrated (ca. 10 s) and then returned to the water bath. The reaction was terminated by adding 1 mL sodium hydroxide solution, after exactly 15 min. The mixture stirred again in the reagent mixer for about 5 s. The solution was filtered through filter papers and poured into 1 cm cuvettes. The absorbance was measured using a spectrophotometer at 620 nm (Biochrom 80-7000-30, Cambridge England) and distilled water was used as a reference. Diastase activity was obtained from the absorbance measurements by using the following equations, and ΔA_620_ was calculated by subtracting the absorbance of the blank from the sample solution [11].

Diastasenumber=28.2xΔA620+2.64 (6)

### Statistical analysis

Data was generated from multiple runs of samples with minimum duplicate measurements. The antioxidant and physicochemical data analyzed by SAS, 2002 using a one-way analysis of variance (ANOVA). The PCA (principal component analysis) was expressed using a biplot graphical method of the multivariate data matrix, which displays the two-dimensional chart that is used to evaluate the relationship between the rows (*Schefflera abyssinica* honey and Polyfloral honey) and columns (different variables of antioxidant and physicochemical properties). PCA was analyzed using XLSTAT 2015.1 statistical software. Correlations among physicochemical and antioxidant properties are done using the Pearson correlation analysis, and was performed by SPSS, Version 20. Results were reported as mean ± SD. Least Significant Difference (LSD) was used for mean separation and ρ<0.05 was considered significant.

## Results and discussion

### Botanical origin

The botanical origin of the honey samples collected from Sheka forest was originated from eight different nectar source plant species. Namely, *Schefflera abyssinica*, *Croton macrostachyus*, *Coffea arabica*, *Vernonia amygdalina*, *Guizotia scabra*, *Eucalyptus spp*, *Syzygium guineense*, and *grass spp*. (Table 1). The level of pollen dominancy for *Schefflera abyssinica* monofloral honey (Fig 1A) range from 76.2 to 85.8% (Table 1). This was in agreement with the report of Karabournioti & Karabagias [27] (68–91%) of Egyptian monofloral honey and Dobre et al. [28] (52–93%) for Romania honey. The polyfloral honey (Fig 1B) collected from Sheka Forest found with variety of nectar contribution range from 2.2% (*Coffea arabica*) to 23.2% (*Schefflera abyssinica*) (Table 1). This was in line with the report of Kruczek & Stacewicz [29] for West Pomeranian honey (8.41–20.67%).

**Table 1:** Pollen count distribution for selected monofloral and polyfloral honey (n = 20).

Honey sample	Plant source (%)							
	*Schefflera abyssinica*	*Croton macrostachyus*	*Coffea arabica*	*Vernonia amygdalia*	*Guizotia scabra*	*Eucalyptus spp*	*Syzygium guineense*	*Grass spp*
S1	79.2	11.6	2.4	1.0	2.6	1.8	-	1.4
S2	76.2	12.4	2	1.6	3.2	2.2	0.4	2
S3	78.4	12.2	1.6	2.0	2.4	1.6	-	1.8
S 4	81.0	11.8	1.2	0.8	1.4	1.6	-	2.2
S5	85.4	8.4	1.2	1.6	0.8	1.2	-	1.4
S6	84.2	10.0	1.6	2.0	1	0.6	-	0.6
S7	83.6	9.2	2.8	1.0	0.6	1.6	-	1.2
S8	85.8	9.8	2.6	1.0	0.6	0.2	-	-
S9	83.6	6.6	3.2	2.2	2.6	1.8	-	-
S10	84.20	9.6	1.0	1.8	1.4	0.8	-	1.2
P1	22.2	19.2	12.4	13.8	5.4	9.0	7.6	10.4
P2	23.2	18.0	2.8	4.4	6.4	9.8	9.4	26
P3	22.2	9.4	13.6	5.6	5.8	19.4	9.0	15.0
P4	21.8	18.0	3.2	5.2	6.8	19.0	9.8	16.2
P5	19.4	14.4	13.6	6.8	6.2	9.2	11.4	19.0
P6	18.8	17.0	12.8	5.8	7.4	10.2	10.8	17.2
P7	19.8	15.6	13.8	5.4	7.2	9.6	11.2	17.4
P8	20.6	16.2	13.2	6.4	7.0	11.2	9.8	15.6
P9	17.6	16.4	2.2	13.2	17.2	9.8	12.8	10.8
P10	16.4	17.8	11.8	3.6	8.2	10.4	14.2	17.6

S = *Schefflera abyssinica* honey; P = Polyfloral honey.

### Antioxidant content

#### Total phenolic content

The total phenols directly related to the antioxidant activity of honey. In addition, phenolic compounds present in honey have used as a floral marker [30]. The mean ± SD of total phenolic content for *Schefflera abyssinica* and polyfloral honey was 75.08 ± 2.40 and 50.65 ± 2.41 mg GAE/100g, respectively (Table 2). A significant difference (p<0.05) was observed among the honey sample. *Schefflera abyssinica* had a higher total phenolic content than polyfloral honey. The variation in the phenolic content could be due to botanical sources of honey [31]. A similar result was reported by do Nascimento et al. [30] for phenolic content of monofloral honey (66.45 ± 15.4) and polyfloral honey (59.37± 13.3 mg GAE/100g).

**Table 2:** Antioxidant content and antioxidant activities (mean ± SD) of *Schefflera abyssinica* and Polyfloral honey (n = 20).

Parameters	Honey samples	
	*Schefflera abyssinica*	Polyfloral honey
Total phenol (mg GAE/100g)	75.08 ± 2.40^a^	50.65 ± 2.41^b^
Total flavonoid (mg CEQ/ 100g)	42.03 ± 1.49^a^	31.07 ± 1.31^b^
DPPH (% inhibition)	44.43 ± 0.97^a^	37.93 ± 1.14^b^
H_2_O_2_ (% inhibition)	78.00 ± 4.82^a^	67.22 ± 2.93^b^
IC_50_ for DPPH (mg/ml)	134.60 ± 8.66^b^	152.84 ± 8.25^a^
IC_50_ for H_2_O_2_ (mg/ml)	36.01 ± 8.01^b^	60.38 ± 10.99 ^a^

Means with different letters in a row were significantly different at P<0.05.

#### Total flavonoids content

One of the main functional components of honey is flavonoids. They can significantly contribute to the total antioxidant activity of honey, which bring beneficial effects for human health [32, 33]. The mean ± SD of flavonoid content for *Schefflera abyssinica* and polyfloral honey was presented in Table 2. The mean ± SD values of flavonoid content were 42.03 ± 1.49 and 31.07 ± 1.31 CEQ /100g for *Schefflera abyssinica* and polyfloral honey, respectively. There was a significant difference (p<0.05) among the honey sample. This was in agreement with the report of Wilczynska [31] for Polish honey (23.81–100 CEQ /100g) and Sime et al. [34] for Ethiopian honey (18 ± 1.5 to 42.2 ± 2.4 CEQ/100 g). Alvarez-Suarez et al. [32] reported (1.09–2.52 mg CE/100 g) for Cuban honey.

Different studies and international organization have approached the possibility to determine the physicochemical properties, geographical and botanical origin of honey; however honey fraud, false and doubtful labeling is severe in the honey industry, worldwide [6]. In this study, we found that antioxidant content regress on the botanical origin of the honey, which used to differentiate honey.

### Antioxidant activity

#### DPPH free radical-scavenging activity

The DPPH assay measures the ability of the honey sample to donate hydrogen to the DPPH radical, which results in a quantitative discoloration of the DPPH reagent, which is related to the antioxidant activity [35]. The DPPH scavenging potential of *Schefflera abyssinica* and polyfloral honey was stated in Table 2. The percentage inhibition of *Schefflera abyssinica* and polyfloral honey was 44.43 ± 0.97 and 37.93 ± 1.14%, respectively. A significant difference (p<0.05) was observed between the honey sample, and *Schefflera abyssinica* had higher DPPH scavenging potential than polyfloral honey.

The current studies for *Schefflera abyssinica* and polyfloral honey were in line with the report of Escuredo et al. [36] for Spain polyfloral honey (35.7 ± 13.0%) and Goslinski et al. [37] for New Zealand Manuka honey (40.0 ± 0.3%). Honey samples originated from Italy were characterized by higher variability of the % DPPH scavenging activity (64.03 ± 7.75%) [38]. The report of Sime et al. [34] for % inhibition of DPPH (18.4 ± 1.6 to 58.9 ± 2.5) was similar with the current study.

#### Hydrogen peroxide (H_2_O_2_) scavenging activity

The Stability and medicinal value of honey are attributable to different factors, which are associated with hydrogen peroxide, low pH, and high Osmolarity [39]. The hydrogen peroxide scavenging potential for *Schefflera abyssinica* and polyfloral honey were 78.00 ± 4.82 and 67.22 ± 2.93% inhibitions, respectively (Table 2). There was a significant difference (p<0.05) between the honey samples. In this study, *Schefflera abyssinica* had higher hydrogen peroxide scavenging activity than polyfloral honey. This was true for Malaysia honey (20.95–76.99%) [40].

Honey consumption, with high H_2_O_2_ scavenging capacity, is highly recommended; which could possibly reduce and/ or abolish the formation of H_2_O_2_ and hence save the body from oxidative damage [41]. *Schefflera abyssinica* and polyfloral honey from Sheka forest had a good ability to scavenge H_2_O_2_ for human health.

#### Inhibitory concentration

The concentration of the material necessary to inhibit 50% of free radical (IC_50_) is important to determine the scavenging activity against the free radical DPPH and H_2_O_2_. The mean ± SD for DPPH IC_50_ value of *Schefflera abyssinica* and polyfloral honey were 134.60 ± 8.66 and 152.84 ± 8.25 mg/ml, respectively (Table 2). A significant difference (p<0.05) was observed among the honey sample. A lower IC_50_ concentration in honey indicates a higher ability to neutralize free radicals [22]. The finding of *Schefflera abyssinica* and polyfloral honey was in agreement with the report of Ferreira et al. [42] for Portuguese honey (84.9–168.9 mg/ml), and Maurya et al. [43] for Czech polyfloral honey (4.4–358 mg/ml). IC_50_ is the amount of antioxidant capacity, which is necessary to decrease the initial concentration by 50% [35]. Temizer et al. [44] had a similar report for IC_50_ value of hydrogen peroxide (122.48–220.46 mg/ml) for polyfloral honey of this finding. do Nascimento et al. [30] reported a lower value of monofloral (65.09± 36.5mg/ml) and polyfloral (82.6 ± 37.6 mg/ml) Brazilian honey than this study.

### Physicochemical properties

#### Moisture content

The mean ± SD of moisture content for *Schefflera abyssinica* and polyfloral honey was presented in Table 3. The moisture content of *Schefflera abyssinica* and polyfloral honey were 19.96 ± 0.26 and 18.90 ± 0.45 g/ 100g, respectively. A significant difference (p<0.05) was observed in moisture content among the honey. This was in line with the report of Belay et al. [45] (20.54 ± 1.28 g/100g). The variation in moisture content between monofloral *(Schefflera abyssinica)* and polyfloral honey among the sample was due to the nectarous plant variation foraged by bees [46].

**Table 3:** Physicochemical properties and sugar profile (mean±SD) of *Schefflera abyssinica* and polyfloral honey (n = 20).

Honey attributes	Honey samples	
	*Schefflera abyssinica*	*Schefflera abyssinica*
Refractive index	1.4866 ± 0.00^b^	1.4892 ± 0.00^a^
Moisture (g/100g)	19.96 ± 0.26^a^	18.90 ± 0.45^b^
Fructose (g/100g)	39.89 ± 1.65^a^	36.33 ± 0.53^b^
Glucose (g/100g)	29.38 ± 1.34^b^	33.94 ± 0.62^a^
Sucrose (g/100g)	0.65 ± 0.17^a^	0.33 ± 0.04^b^
Turanose (g/100g)	ND	ND
Maltose (g/100g)	ND	ND
Fructose: Glucose ratio	1.36 ± 0.06^a^	1.07 ± 0.02^b^
Reducing sugar (g/100g)	69.27 ± 2.54^b^	70.27 ± 1.04^a^
Optical density	0.16 ± 0.01^b^	0.26 ± 0.03^a^
Ash (g/100g)	0.28 ± 0.04^b^	0.41 ± 0.05^a^
Electrical Conductivity (mS/cm)	0.33 ± 0.05^b^	0.42 ± 0.02^a^
pH	3.80 ± 0.07^b^	4.01 ± 0.08^a^
Free Acidity (meq/Kg)	23.68 ± 7.28^a^	24.34 ± 3.64^a^
Protein (g/100g)	0.43 ± 0.05^b^	0.51 ± 0.07^a^
Hydroxymethylfurfural(mg/Kg)	6.12 ± 2.14^a^	4.37 ± 1.83^b^
Color (pfund)	53.10 ± 1.83^b^	130.58 ± 0.75^a^
Total soluble solids (Brix)	78.44 ± 0.22^b^	79.53 ± 0.44^a^
Diastase (Schade units)	4.10 ± 0.30^b^	5.21 ± 0.17^a^
Refractive index	1.4866 ± 0.00^b^	1.4892 ± 0.00^a^

Means with different letters in a row were significantly different at P<0.05; ND = Not detected.

Moisture is one of the most important quality parameter of honey. The amount of water present in honey determines its stability against fermentation and granulation [47]. High moisture could increase honey fermentation by certain osmotolerant yeasts [47]. The moisture content of all samples analyzed was within the acceptable range of international standard of Codex Alimentarius (not more than 20 g/100g) [12].

The mean ± SD values of refractive index for *Schefflera abyssinica* and polyfloral honey were 1.4866 ± 0 and 1.4892 ± 0 respectively (Table 3). There was a significant difference (p<0.05) observed in the refractive index among the honey samples. This was in agreement with the report of Belay et al. [45] (1.4845) and Balasubramanyam [48] (1.4956).

#### Optical density

The mean ± SD of optical density for *Schefflera abyssinica* and polyfloral honey was 0. 16 ± 0.01 and 0.26 ± 0.03, respectively (Table 3). A significance difference (p<0.05) was observed among *Schefflera abyssinica* and polyfloral honey sample. The optical density of the current study was in agreement with the report of Owayss [49] for Libyan monofloral honey (0.13). EI Borai et al. [50] reported optical density of Egyptian honey (0.5–1.05) and Balasubramanyam [48] for Karnataka honey (0.61–0.67), which was higher than the optical density of *Schefflera abyssinica* and polyfloral honey. This could be due to moisture content and the floral origin of honey [24].

#### Sugar profile

**Fig 2:**
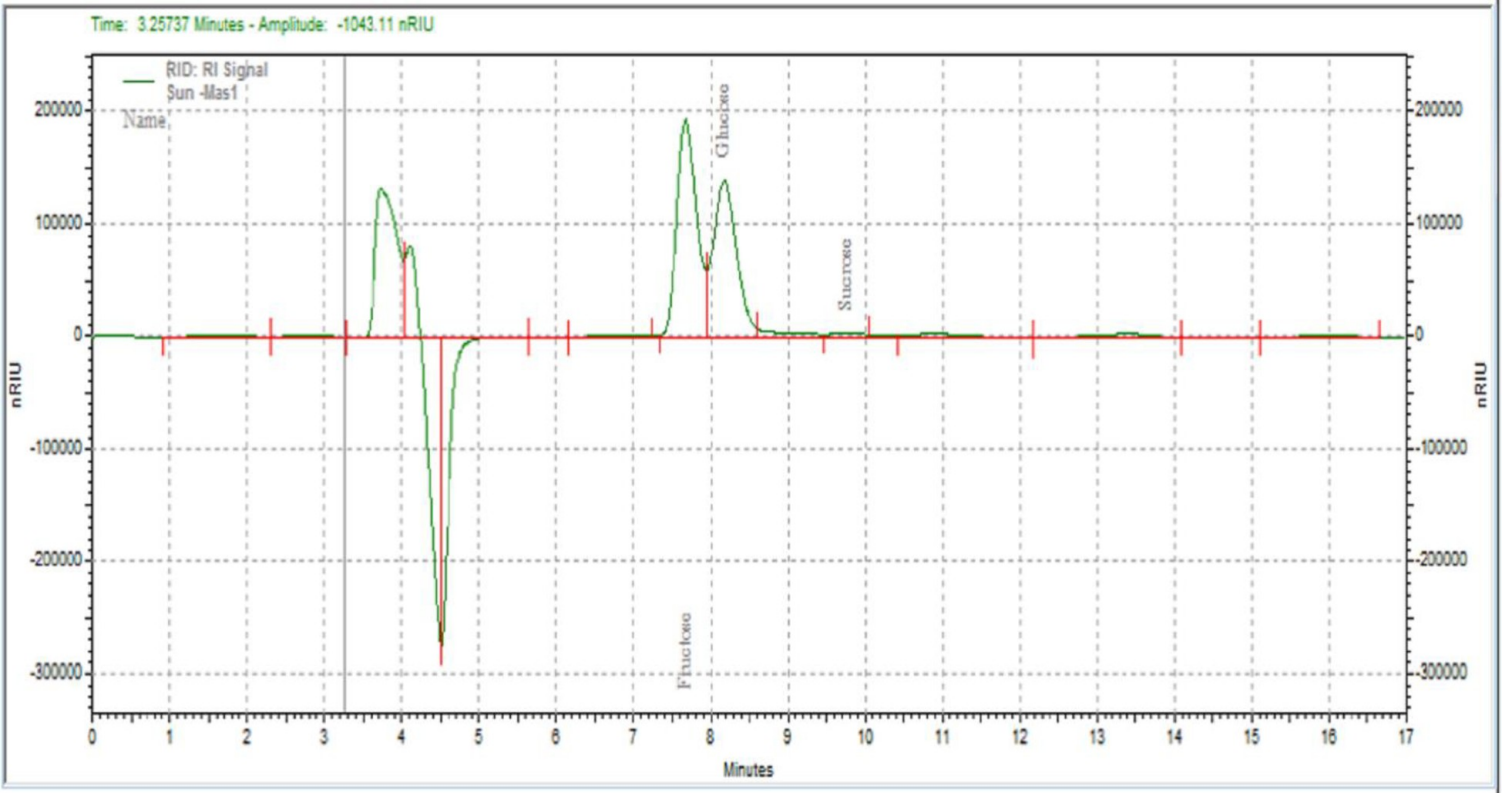
HPLC chromatogram for sugar profile of honey sample.

HPLC chromatogram (where retention time of Fructose = 7.465, Glucose = 8.106 and Sucrose = 9. 527 min) for the sugar profile of the analyzed honey sample was presented in Fig 2. The sugar content of the sample was calculated by comparing the corresponding peak areas of the sample and those of the pure standard solutions.

Sugars are the major component of honey and responsible for properties such as energy value, viscosity, hygroscopicity, and granulation of honey [51]. The result of sugars, namely fructose, glucose, sucrose, maltose, and turanose are presented in Table 3. The mean ± SD value of fructose content was 39.89 ± 1.65 and 36.33 ± 0.53 g/100g for *Schefflera abyssinica* and polyfloral honey, respectively. A significant difference (p < 0.05) was observed among the honey samples. The fructose content of *Schefflera abyssinica* was in line with Belay et al. [45] (38.81 ± 1.18 g/ 100g) and Escuredo et al. [36] (38.60 g/100g) report for monofloral honey. Temizer et al. [44] reported fructose content of polyfloral honey (36. 58 g/100g), which was in agreement with the current study of polyfloral honey.

do Nascimento et al. [30] reported fructose content for Brazilian monofloral honey (38.70 ± 1.24 g /100g) and Chakir et al. [52] reported for Morocco monofloral honey (39.37 ± 0.60 g/100g), which was similar to the current finding. The fructose content of polyfloral honey for this study was in agreement with a report of Habib et al. [53] (36.82 ± 0.07 g/100g) for UAE polyfloral honey and do Nascimento et al. [30] (37.70 ± 1.46 g/ 100g) of Brazilian polyfloral honey.

Mean ± SD values of glucose content for *Schefflera abyssinica* and polyfloral honey were 29.38 ± 1.34 and 33.94 ± 0.62 g /100g, respectively (Table 3). There was a statistically significant difference (p<0.05) between the honey samples. The glucose content of *Schefflera abyssinica* honey of the current study was similar to Nguyen et al. [54] report of New Zealand honey (28.9 ± 2.0 g/100g), Belay et al. [45] (30.55 ± 2.69 g /100g), and Hagr et al. [55] (31.7 ± 0.68 g /100g).

The concentration of fructose and glucose and their ratio are useful indicators for the classification and assessment of the rate of crystallization in honey [24]. The ratio of f:g (fructose:glucose) for *Schefflera abyssinica* and polyfloral honey was 1.36 ± 0.06 and 1.07 ± 0.02, respectively (Table 3). A significant difference (p<0.05) was observed among the honey sample in f:g, which was in agreement with the report of do Nascimento et al. [30] for monofloral (1.2 ± 0.1) and polyfloral (1.1 ± 0.13) honey. Serem & Bester [56] of South Africa (0.85–1.31) and Al et al. [57] Romania honey (0.81–1.4) was also reported similar f:g ratio with this finding.

In both honey samples, glucose content was lower than the fructose content, which is true for the majority of honey. Exceptionally, *Brassica napus* honey had high glucose than fructose [58]. The sum of Fructose + glucose, largely consider as reducing sugar established by Codex Alimentarius as standard (not less than 60 g/100 g) [11]. Accordingly, *Schefflera abyssinica* (69.27 ± 2.54) and polyfloral honey (70.27 ± 1.04) were met the standard set by Codex Alimentarius, European Union and Ethiopian standards [12–14].

Sucrose is an essential sugar, mostly used as a quality standard. The contribution of sucrose to total sugar in honey can be increased, if honey is harvested before ripening [45]. The mean ± SD of sucrose content for *Schefflera abyssinica* and polyfloral honey were 0.65 ± 0.17 and 0.33 ± 0.04 g/100g, respectively (Table 3). A statistically significant difference (p<0.05) was observed between the mean values of sucrose contents among the honey sample. According to Erturk et al. [59], the sucrose content of monofloral and polyfloral honey were 0.97–3.13 and 0.98–2.01 g/100g, respectively, and the report of Ouchemoukh et al. [60] (0.23–3.41 g.100g) of sucrose for Algerian honey, were within the range of the present study.

The Codex Alimentarius, European Union, and Ethiopian standard allows a maximum sucrose content of 5 g/100g in honey. The sucrose content is used to detect improper handling of honey. High levels of sucrose related to inadequate maturation or prolonged sucrose syrups feeding of bees and early harvesting [56]. In this study, a lower level of sucrose was observed compared to the maximum limit. This indicates the strong culture of Sheka forest beekeepers to harvest a matured honey.

Maltose and turanose content of *Schefflera abyssinica* and polyfloral honey was determined. However, both maltose and turanose was below the detection limit for the applied method in *Schefflera abyssinica* and polyfloral honey samples. This result was consistent with the report of Sousa et al. [61] and Ruoff et al. [62]. The variation in sugar composition might be due to the botanical origin of honeybees [51, 52].

#### Ash content

The mean ± SD of ash content was 0.28 ± 0.04 and 0.41 ± 0.05 g/100g for *Schefflera abyssinica* and polyfloral honey, respectively (Table 3). A significant difference (p<0.05) was observed in ash content among the honey samples. These values were comparable with monofloral (0.33 g/100g) and polyfloral honey (0.42 g /100g) reported by Erturk et al. [59].

The ash content of this finding was higher than the value reported by Abdulkhaliq & Swaileh [47] for Palestine polyfloral honey (0.03–0.21 g /100g). The mean ash content of Thailand monofloral honey was 0.16 g/ 100g [46], which was lower than this finding. The variation could be due to the difference in botanical origin, geographical location, and beekeeping practice [63].

Ash content reflects the chemical composition of the plant from which the honeybees collect their food [59]. Both, *Schefflera abyssinica* and polyfloral, honeys of this report met the standards proposed by the Codex Alimentarius and Ethiopian standard (not more than 0.60 g/100g) in ash content. In addition, these values showed all the honey samples of the Sheka Forest originated from a nectar source plant [12, 14].

#### Electrical conductivity

The mean ± SD of electrical conductivity for *Schefflera abyssinica* and polyfloral honey of this finding were 0.33 ± 0.05 and 0.42 ± 0.02 mS/cm, respectively (Table 3). A significance difference (p<0.05) was observed among the honey sample. The electrical conductivity of *Schefflera abyssinica* was similar to the finding of Belay et al. [45] (0.32 ± 0.02 mS/cm), and higher than the value reported by Ruoff et al. [62] (0.10–0.27 mS/cm) for Acacia honey, and lower than Eucalyptus honey found by do Nascimento et al. [30] (0.65 ± 0.15 mS/cm).

Electrical conductivity is used as an indicator for quality control of honey that can be used to distinguish floral honey from honeydew honey [1]. The electrical conductivity of honey in this study was within the recommendation of Codex Alimentarius and Ethiopian standard (not more than 0.8 mS/cm) [12, 13]; and both *Schefflera abyssinica* and polyfloral honey were originated from nectar source plant species. According to Karabagias et al. [1], variations in electrical conductivity of honey samples were linked to variations in the botanical origin of honey.

#### pH and free acidity

The mean ± SD value of pH for *Schefflera abyssinica* and polyfloral honey were 3.80 ± 0.07 and 4.01 ± 0.08, respectively (Table 3). A significant difference (p< 0.05) was observed among the honey sample in pH. The pH value of *Schefflera abyssinica* in the current study was in agreement with Belay et al. [45] (3.77 ± 0.23), and Temizer et al. [44] (4.08). pH limit has not yet been described by the regulatory committees. However, pH level between 3.2 and 4.5 considered as a natural acidity of the honey, which is known in inhibiting the growth of micro-organisms [1]. Accordingly, *Schefflera abyssinica* and polyfloral honey have natural acidity to inhibit the growth of microorganism.

The mean ± SD of free acidity for *Schefflera abyssinica* and polyfloral honey were 23.68 ± 7.28 and 24.34 ± 3.64 meq/kg, respectively (Table 3). There was no significant difference (p > 0.05) among the honey sample in free acidity. The free acidity of *Schefflera abyssinica* in this study was in agreement with the report of Belay et al. [45], (23.90 ± 1.85 meq/ kg). Codex Alimentarius [12] permits a maximum value of 50 meq/ kg free acidity. Both, *Schefflera abyssinica* and polyfloral honey of the current study met the standard and possibly taken as fresh honey. Higher free acid can be an indicator of the fermentation of sugars into organic acids. The presence of different organic acids, storage conditions, geographical origin, mineral content, and harvest season can affect the honey acidity [64].

#### Protein content

The mean ± SD of protein content for *Schefflera abyssinica* and polyfloral honey were 0.43 ± 0.05 and 0.51 ± 0.07 g/100g, respectively (Table 3). A significant difference (p < 0.05) was observed among the honey samples. Escuredo et al. [36] reported the protein content for Spanish monofloral (0.59 ± 0.07 g/100g) and polyfloral (0.70 ± 0.23 g / 100g) honey, which was higher than the current study. The protein content of this finding was similar to the value reported by Anklam [65] (0.20 and 0.49 g/100g) and Nguyen et al. [54] New Zealand Manuka honey (0.13 g /100g).

#### Hydroxymethylfurfural

Hydroxymethylfurfural (5-HMF) content is used as an indicator of heat processing and/or storage time of honey. It is formed by the decomposition of fructose in the presence of acids [66]. The HMF content of *Schefflera abyssinica* and polyfloral honey were 6.12 ± 2.14 and 4.37 ± 1.83 mg/kg, respectively (Table 3). There was a significant difference (p<0.05) in HMF content among the honey samples. Belay et al. [15], reported the HMF value of the Harenna forest honey (0.00 to 1.71 mg/kg), which was lower than this study; contrary to this, a higher value of HMF reported by Kowalsk [67] for Acacia (22.36 mg/ kg) and Kamal et al. [68] for polyfloral (16.6–42.9 mg/kg) honey.

According to Codex Alimentarius [12], honey with HMF more than 80 mg/kg for tropical climate indicates heating. EU and Ethiopian standards had a maximum limit of 40 mg/Kg. Accordingly, all the honey samples satisfy the Codex, EU, and Ethiopian standard​;​ and more than 150 mg/kg is an indication of adulteration with invert sugar. *Schefflera abyssinica* and polyfloral honey from Sheka forest considered as fresh honey, which indicate proper honey handling practices.

#### Color

Color is one of the honey attribute, used by consumers for quality appreciation and acceptability [69]. The pfund value of *Schefflera abyssinica* and polyfloral honey were 53.10 ± 1.83 mm and 130.58 ± 0.75 mm, respectively (Table 3). A significance difference (p < 0.05) was observed among the honey sample. El Sohaimy et al. [24] reported the pfund value of Yemeni monofloral honey (56.40 ± 2.32 mm), which was in agreement with *Schefflera abyssinica* honey of this study, while Escuredo et al. [36], found higher pfund value (73 ± 1.3 mm) of Spanish eucalyptus honey. The pfund value of Brazilian polyfloral (151.08 mm) reported by Pontis et al. [70], was higher than the polyfloral honey of Sheka forest.

The color of the analyzed honey samples varied between extra light amber (*Schefflera abyssinica)* and dark amber (polyfloral honey). Honey colour depends on various factors, and minerals content is the major factor influencing honey color. Light-coloured honey usually have low ash contents, while dark-coloured honey generally have higher ash contents [71], which was consistent with the finding of this study.

#### Total soluble solids (Brix)

The mean ± SD of °Brix values, which represent the total soluble solids, was 78.44 ± 0.22 and 79.53 ± 0.44 for *Schefflera abyssinica* and polyfloral honey, respectively (Table 3). A significant difference (p<0.05) was observed among the honey samples. Oroian et al. [72] reported the ^0^Brix value for Romania monofloral (78.2–84.1^0^Brix) and polyfloral (76.3–81.7 ^0^Brix) honey, which was consistent with the current study, while Al-Farsi et al. [22] found a Brix value (82.0–82.5 ^0^Brix) of Omani honey.

#### Diastase activity

Diastase is widely used as an indicator of honey freshness. It decreases due to excessive storage and heating of honey [46]. Some honey types have also low diastase, naturally [73]. Diastase activities of *Schefflera abyssinica* and polyfloral honey of the current study were 4.10 ± 0.30 and 5.21 ± 0.17 Schade units, respectively (Table 3). A significant difference (p<0.05) was observed among the honey sample. Belay et al. [73] reported the diastase activity of monofloral honey (4.94 ± 0.66 Schade units), which was in agreement with this finding. The difference in diastase activity depends on the nectar collection period, the physiological period of the colony, and pollen consumption [74]. Diastase activity is important to detect and predict honey age/freshness, storage time, and overheating of honey [69].

The reports from the revised Codex standard for honey stated that diastase activity of honey is > 3 Schade unit, when HMF is less than 15 mg/kg; otherwise, it is 8 Schade unit [12]. Accordingly, both *Schefflera abyssinica* and polyfloral honey met the standard (> 3 Schade unit) proposed by Codex Alimentarius.

### Correlation between physicochemical and antioxidant properties of honey

Pearson Correlation for the physicochemical and antioxidant properties of *Schefflera abyssinica* and polyfloral honey was presented in Table 4. The measurement of refractive index and moisture content was negatively correlated (r = -1). This is based on the principle that light move faster through honey that has few solids than many solid; accordingly, the refractive index increases with solid content [75]. Moisture and sucrose were positively correlated (r = 0.663). The moisture content of honey is widely related to the harvesting season and the level of maturity, which was explained by sucrose content. Accordingly, both moisture and sucrose inversely related to ripened honey [72]. pH and color were also correlated (r = 0.809) for *Schefflera abyssinica* and polyfloral honey, which was also explained by A-Rahaman et al [76] (r = 0.971) for Malaysia honey.

**Table 4:** Pearson correlation matrix among physicochemical and antioxidant properties of *Schefflera abyssinica* and polyfloral honey.

Variables	Moisture	OD	pH	Ash	EC	Color	Fructose	Glucose	Sucrose	Brix	TPC	TFC	DPPH	H_2_O_2_
RI	-1.**	.496	.520	.646	.590	. 582	-.573	.178	-.563	.997*	-.283	-.228	-.273	-.186
Moisture		-.496	-.520	-.646	-.590	-.582	. 573	-.718	.663*	-.997*	.283	.228	.273	.186
OD			.594	. 478	.576	.887*	-.581	. 480	-.490	. 651	-.489	-.287	-.478	-.467
pH				.467	.560	.809*	-.475	.396	-.433	.541	-.578	-.476	-.580	-.285
Ash					.868*	.848*	-.634	.489	-.371	. 569	.827*	.800*	.746*	.678*
EC						. 280	-.501	.189	-.362	.608	.755*	.765*	.720*	.809*
Color							-.481	.199	-.797	.468	981*	970*	952*	.817*
Fructose								-.563	.385	-.572	.580	.577	.475	.540
Glucose									-.671*	.560	-.487	-.690	-.386	-.189
Sucrose										-.654	.483	.117	.447	.544
Brix											.583	.338	.557	.564
TPC												.934*	.918*	.738*
TFC													.934*	.832*
DPPH														.805*

DPPH = 1,1-diphenyl-2-picrylhydrazyl scavenging activity; EC = electrical density; H2O2 = hydrogen peroxide scavenging activity; RI = refractive index; OD = optical density; TFC = total flavonoid content; TPC = total phenolic content.

* Correlation is significant at p< 0.01.

A correlation (r = 0.868) was found between ash and electrical conductivity, and this was reported by Belay et al. [45] (r = 0.689) for Ethiopian monofloral honey. There were significant (p< 0.01) correlation (r = 0.848) between ash and color. Ash, which relates to the place where the plant grew, had a major influence on the colour of honey. Light-coloured honey usually has low ash, while dark-coloured honey contains high ash [71]. An inverse relation was also found between glucose and sucrose (r = -0.671). The conversation of sucrose, by invertase enzyme, into monosaccharide sugars is a good indicator of honey ripeness, which decreases sucrose level, and inversely increases glucose content [70].

The correlation analysis of the current study indicated that moisture and ^0^Brix of Sheka Forest honey had a significant (p<0.01) correlation (r = 0.997), which was in agreement with the report of Anupama et al. [77] (r = -0.990) for Indian honey. The association showed that the value of ^0^Brix can be an indirect indicator of the moisture content of honey. A significant (p< 0.01) correlation (r = 0.887) between optical density and color of honey samples were also observed in the Sheka Forest honey, which was in line with the report of Balasubramanyam [48] that state lighter honey has less optical density compared to dark honey.

The total phenolic and total flavonoid content of the Sheka Forest honey significantly correlated (p<0.01) with the ash content at r = 0.827 and r = 0.80, respectively (Table 4). The ash/mineral contents of the honey contribute to the color of the honey, which consequently had an impact on the photometric measurement of total phenolic and total flavonoids content of the honey [1, 69, 78]. The relation of ash with total phenolic (r^2^ = 0.70) and total flavonoids (r^2^ = 0.80) contents of Venezuelan honey was in line with this finding [79].

The spectrophotometric value of the color of Sheka Forest honey correlates with total phenolic content, total flavonoid content, DPPH, and H_2_O_2_ values, at r values of 0.981, 0.970, 0.952 and 0.817, respectively (Table 4). Similar results were also reported by Bertoncelj et al. [80] for Italian honey (r^2^ = 0.837) and Moniruzzaman et al. [81] Malaysian honey (r^2^ = 0.933). According to Khalil et al. [82], the color had a role in the antioxidant activities of honey samples, and the color value increased with the phenolic and flavonoid contents of honey.

A significant (p<0.01) correlation was found between total phenolic content and DPPH activity (r = 0.918), and total flavonoids content and DPPH activity (r = 0. 934) of Sheka Forest honey. This was in line with the report of Islam et al. [83] (r = 0.869) and Ruiz-Navajas et al. [84] (r = 0. 0.92) for Mexican honey.

**Fig 3:**
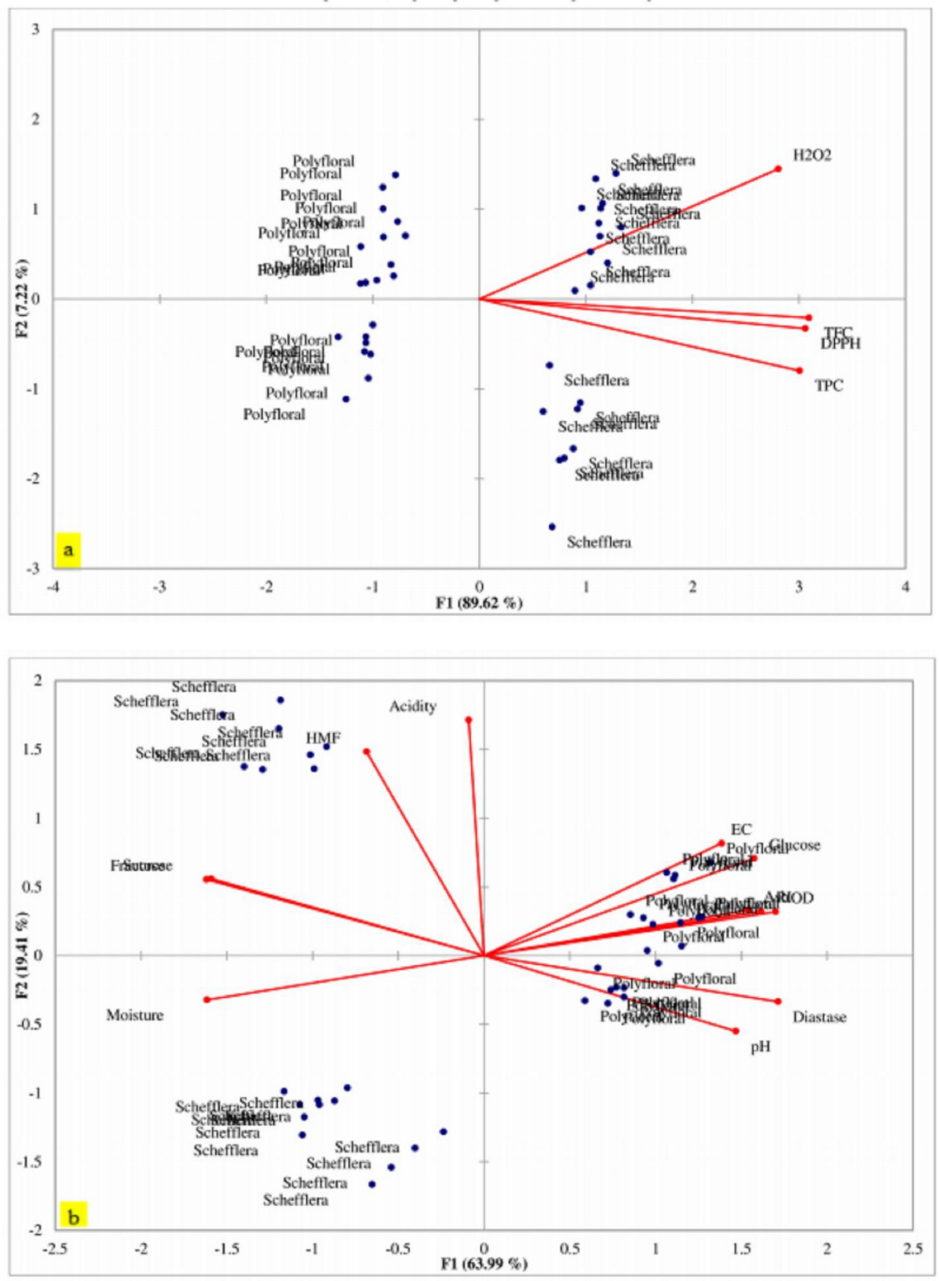
Principal component analysis predictive biplots of botanical origin over antioxidant (a) and physicochemical (b) properties. The degree of proximity between variables and the narrower angle between diagonal lines indicated a strong association. TPC = Total phenolic content; TFC = Total flavonoid content; DPPH = 1, 1-diphenyl-2-picrylhydrazyl scavenging activity; H_2_O_2_; hydrogen peroxide scavenging activity; RI = refractive index; OD = optical density; HMF = hydroxymethylfurfural; EC = Electrical conductivity.

A multivariate analysis was performed to look for main data structures of *Schefflera abyssinica* and polyfloral honey and the possible trends, and the degree of variations observed between variables (Fig 3A and 3B). PCA with predictive biplots was chosen to carryout interpretations based on respective associations. A straight line was drawn from a sample point to a variable axis; and the smaller the angle and degree of the proximity between variables indicated a strong association. Fig 3A described 96.84% of the variation in the data set, in which PC_1_ explained 89.62% of the variability and PC_2_ explained 7.22%. The PCA biplots in Fig 3A indicated the existence of two important data structures, namely *Schefflera abyssinica* on the right quadrants and polyfloral honey on the left. In addition, Fig 3A inferred an inferior association between these two main structures. *Schefflera abyssinica* associated with the antioxidant variables of total phenolic, total flavonoids, DPPH, and H_2_O_2_; on the other hand, polyfloral honey far to associate with total phenolic, total flavonoids, DPPH, and H_2_O_2_. Likewise, floral honey sources can essentially be differentiated by antioxidant patterns, and the distribution of pollen associated with total phenolic, total flavonoids, DPPH, and H_2_O_2_. There was a clear separation of the samples by floral origin (Fig 3A). *Schefflera abyssinica* honey differed from the polyfloral types of honey and clustered on the positive side. In contrast, polyfloral honey was on the negative side (Fig 3A). This showed that *Schefflera abyssinica* and polyfloral honey clearly separated based on antioxidant properties. Moreover, this approach could give *Schefflera abyssinica* honey a precise territory brand and a guarantee of geographical origin and traceability. This can possibly differentiate the fraud and doubtful labeling of *Schefflera abyssinica* and polyfloral honey collected from Sheka Forest. The report of Kivrak et al. [85] was in agreement with this finding. The monofloral honey on the right side of the positive value of PC_1_ was trees of *Schefflera abyssinica*. This tree is indigenous bee tree species promising for honey production. It is one of the most important honey trees of the Sheka forest, which has abundant nectar and pollen, suitable for the honeybee. The tree used to produce large quantities of light and pure white honey, which has a higher demand in the market [86, 87].

The association between botanical origin and physicochemical properties was sketched on the PCA biplots, and the principal components explained 83.40% of the variation in the data set. PC_1_ explained 63.99% of the variability and PC_2_ explained 19.41% (Fig 1B). The components were categorized into two main groups, in relation to the physicochemical characteristics of honey. Electrical conductivity, glucose content, ash, refractive index, optical density, diastase activity, and pH associated with each other and with polyfloral honey. On the other side, acidity, hydroxymethylfurfural, fructose, sucrose, and moisture content associated with each other, and explained by *Schefflera abyssinica*.

## Conclusion

In this study, the botanical origin, antioxidant, and physicochemical properties of *Schefflera abyssinica* and polyfloral honey from Sheka Forest, Ethiopia was investigated. Floral origin and antioxidant properties of honey were strongly associated, and can be used to differentiate *Schefflera abyssinica* and polyfloral honey. A positive correlation was found between the ash content with total phenolic and total flavonoid contents. The color of Sheka Forest honey was also correlated with the phenolic content, flavonoid content, DPPH, and H_2_O_2_ values. A correlation was also found between phenolic and flavonoid contents with antioxidant activities (DPPH and H_2_O_2_); this indicated that total flavonoids and total phenolic content could be responsible for the antioxidant activities of honey. *Schefflera abyssinica* and polyfloral honey were found to meet the recommended national and international standards. A PCA with predictive biplots confirmed the existence of significant associations, and the botanical origin of the honey significantly differentiated by antioxidant properties, and the distribution of *Schefflera abyssinica* pollen linked with total phenolic, total flavonoids, DPPH, and H_2_O_2_. The result of the study showed that floral origin had an effect on the antioxidant and physicochemical properties of honey.

## Supporting information

S1 Data (DOCX)
