# Supplementary material for: Melissopalynology and antioxidant properties used to differentiate Schefflera abyssinica and polyfloral honey
Source: PLoS One. 2020 Oct 28;15(10):e0240868. doi: 10.1371/journal.pone.0240868 (PMC7592792; doi:10.1371/journal.pone.0240868)
Supplement: S1 Data — (DOCX) [file pone.0240868.s001.docx]

Appendix Table 1. Pollen count for monofloral and polyfloral honey.

|  | *Schefflera* | | | | *Croton* | | | | *Coffea* | | | | *Vernonia* | | | | *Guizotia* | | | | *Eucalyptus* | | | | *Syzygium* | | | | *Grass* | | |
| --- | --- | --- | --- | --- | --- | --- | --- | --- | --- | --- | --- | --- | --- | --- | --- | --- | --- | --- | --- | --- | --- | --- | --- | --- | --- | --- | --- | --- | --- | --- | --- |
|  | Count  1 | Count  2 | Mean | Count  1 | | Count  2 | Mean | Count  1 | | Count  2 | Mean | Count  1 | | Count  2 | Mean | Count  1 | | Count  2 | Mean | Count  1 | | Count  2 | Mean | Count  1 | | Count  2 | Mean | Count  1 | | Count  2 | Mean |
| S | 344 | 448 | 396 | 50 | | 66 | 58 | 14 | | 10 | 12 | 5 | | 5 | 5 | 13 | | 13 | 13 | 9 | | 9 | 9 | 0 | | 0 | 0 | 7 | | 7 | 7 |
| S | 389 | 373 | 381 | 67 | | 57 | 62 | 8 | | 12 | 10 | 8 | | 8 | 8 | 16 | | 16 | 16 | 11 | | 11 | 11 | 2 | | 2 | 2 | 10 | | 10 | 10 |
| S | 343 | 441 | 392 | 65 | | 57 | 61 | 7 | | 9 | 8 | 11 | | 9 | 10 | 12 | | 12 | 12 | 8 | | 8 | 8 | 0 | | 0 | 0 | 9 | | 9 | 9 |
| S | 412 | 398 | 405 | 56 | | 62 | 59 | 6 | | 6 | 6 | 4 | | 4 | 4 | 7 | | 7 | 7 | 8 | | 8 | 8 | 0 | | 0 | 0 | 11 | | 11 | 11 |
| S | 403 | 451 | 427 | 44 | | 40 | 42 | 6 | | 6 | 6 | 8 | | 8 | 8 | 4 | | 4 | 4 | 6 | | 6 | 6 | 0 | | 0 | 0 | 7 | | 7 | 7 |
| S | 488 | 354 | 421 | 47 | | 53 | 50 | 8 | | 8 | 8 | 10 | | 10 | 10 | 5 | | 5 | 5 | 3 | | 3 | 3 | 0 | | 0 | 0 | 3 | | 3 | 3 |
| S | 452 | 384 | 418 | 52 | | 40 | 46 | 12 | | 16 | 14 | 5 | | 5 | 5 | 3 | | 3 | 3 | 8 | | 8 | 8 | 0 | | 0 | 0 | 6 | | 6 | 6 |
| S | 449 | 409 | 429 | 65 | | 33 | 49 | 13 | | 13 | 13 | 5 | | 5 | 5 | 3 | | 3 | 3 | 0 | | 2 | 1 | 0 | | 0 | 0 | 0 | | 0 | 0 |
| S | 473 | 363 | 418 | 37 | | 29 | 33 | 14 | | 18 | 16 | 11 | | 11 | 11 | 13 | | 13 | 13 | 9 | | 9 | 9 | 0 | | 0 | 0 | 0 | | 0 | 0 |
| S | 488 | 354 | 421 | 54 | | 42 | 48 | 5 | | 5 | 5 | 9 | | 9 | 9 | 19 | | -5 | 7 | 4 | | 4 | 4 | 0 | | 0 | 0 | 6 | | 6 | 6 |
| P | 156 | 66 | 111 | 99 | | 93 | 96 | 58 | | 66 | 62 | 72 | | 66 | 69 | 23 | | 31 | 27 | 49 | | 41 | 45 | 42 | | 34 | 38 | 55 | | 49 | 52 |
| P | 132 | 100 | 116 | 98 | | 82 | 90 | 14 | | 14 | 14 | 24 | | 20 | 22 | 33 | | 31 | 32 | 44 | | 54 | 49 | 49 | | 45 | 47 | 143 | | 117 | 130 |
| P | 122 | 100 | 111 | 49 | | 45 | 47 | 71 | | 65 | 68 | 32 | | 24 | 28 | 28 | | 30 | 29 | 102 | | 92 | 97 | 48 | | 42 | 45 | 76 | | 74 | 75 |
| P | 121 | 97 | 109 | 99 | | 81 | 90 | 14 | | 18 | 16 | 24 | | 28 | 26 | 36 | | 32 | 34 | 98 | | 92 | 95 | 44 | | 54 | 49 | 89 | | 73 | 81 |
| P | 112 | 82 | 97 | 73 | | 71 | 72 | 76 | | 60 | 68 | 32 | | 36 | 34 | 32 | | 30 | 31 | 44 | | 48 | 46 | 65 | | 49 | 57 | 97 | | 93 | 95 |
| P | 106 | 82 | 94 | 81 | | 89 | 85 | 65 | | 63 | 64 | 28 | | 30 | 29 | 38 | | 36 | 37 | 59 | | 43 | 51 | 64 | | 44 | 54 | 88 | | 84 | 86 |
| P | 112 | 86 | 99 | 89 | | 67 | 78 | 71 | | 67 | 69 | 22 | | 32 | 27 | 39 | | 33 | 36 | 49 | | 47 | 48 | 58 | | 54 | 56 | 98 | | 76 | 87 |
| P | 98 | 108 | 103 | 85 | | 77 | 81 | 65 | | 67 | 66 | 32 | | 32 | 32 | 34 | | 36 | 35 | 55 | | 57 | 56 | 54 | | 44 | 49 | 89 | | 67 | 78 |
| P | 82 | 94 | 88 | 88 | | 76 | 82 | 11 | | 11 | 11 | 58 | | 74 | 66 | 90 | | 82 | 86 | 56 | | 42 | 49 | 66 | | 62 | 64 | 61 | | 47 | 54 |
| P | 78 | 86 | 82 | 94 | | 84 | 89 | 53 | | 65 | 59 | 18 | | 18 | 18 | 41 | | 41 | 41 | 49 | | 55 | 52 | 87 | | 55 | 71 | 81 | | 95 | 88 |

S: *Schefflera abyssinica*; P: Polyfloral

Appendix Table 2. **Antioxidant properties of *Schefflera abyssinica*** and Polyfloral honey.

| **Honey** | **Phenol**  **(mg GAE/100g)** | **Flavonoid**  **(mg CEQ 100g)** | **DPPH**  **(% inhibition )** | **H_2_O_2_**  **(% inhibition)** | **IC_50_ DPPH (mg/ml)** | **IC_50_ H_2_O_2_ (mg/ml)** |
| --- | --- | --- | --- | --- | --- | --- |
| *Schefflera* | 78.27 | 40.18 | 45.13 | 72.10 | 126.54 | 39.67 |
| *Schefflera* | 76.68 | 41.22 | 45.47 | 74.25 | 124.28 | 37.24 |
| *Schefflera* | 78.27 | 41.44 | 43.99 | 71.72 | 132.93 | 45.19 |
| *Schefflera* | 76.79 | 41.16 | 45.13 | 68.62 | 131.57 | 40.38 |
| *Schefflera* | 76.21 | 39.58 | 43.27 | 72.74 | 136.68 | 31.53 |
| *Schefflera* | 78.85 | 41.03 | 45.02 | 74.95 | 133.72 | 35.01 |
| *Schefflera* | 74.00 | 42.04 | 42.38 | 74.26 | 135.15 | 32.31 |
| *Schefflera* | 76.52 | 42.51 | 45.10 | 72.41 | 135.89 | 31.25 |
| *Schefflera* | 76.68 | 41.14 | 43.12 | 78.71 | 137.53 | 26.30 |
| *Schefflera* | 74.50 | 42.04 | 44.17 | 80.50 | 134.61 | 29.96 |
| *Schefflera* | 72.52 | 43.60 | 44.15 | 82.20 | 116.64 | 28.72 |
| *Schefflera* | 73.57 | 42.32 | 44.94 | 79.05 | 118.78 | 33.41 |
| *Schefflera* | 76.75 | 42.49 | 43.45 | 83.18 | 140.44 | 26.66 |
| *Schefflera* | 76.40 | 42.45 | 45.16 | 81.05 | 137.49 | 24.71 |
| *Schefflera* | 72.41 | 42.51 | 42.95 | 81.46 | 139.89 | 29.96 |
| *Schefflera* | 71.51 | 40.74 | 45.11 | 83.23 | 140.01 | 28.13 |
| *Schefflera* | 72.21 | 43.60 | 45.13 | 84.18 | 152.10 | 46.51 |
| *Schefflera* | 71.55 | 45.36 | 45.83 | 82.08 | 150.12 | 50.32 |
| *Schefflera* | 72.29 | 44.79 | 43.99 | 80.97 | 135.36 | 50.63 |
| *Schefflera* | 75.59 | 40.36 | 45.05 | 82.26 | 132.24 | 52.34 |
| Polyfloral | 51.13 | 32.52 | 38.64 | 68.40 | 163.93 | 49.09 |
| Polyfloral | 50.28 | 30.75 | 38.29 | 72.41 | 160.31 | 52.67 |
| Polyfloral | 48.18 | 29.31 | 39.21 | 70.37 | 145.38 | 46.12 |
| Polyfloral | 49.50 | 30.61 | 37.65 | 66.85 | 144.13 | 44.93 |
| Polyfloral | 51.13 | 29.52 | 39.71 | 63.60 | 143.64 | 52.47 |
| Polyfloral | 49.03 | 31.32 | 38.54 | 64.41 | 140.72 | 48.23 |
| Polyfloral | 47.25 | 33.13 | 38.03 | 66.90 | 150.20 | 54.27 |
| Polyfloral | 51.13 | 31.52 | 37.50 | 69.54 | 151.67 | 56.88 |
| Polyfloral | 53.34 | 30.49 | 37.56 | 64.70 | 162.79 | 59.43 |
| Polyfloral | 52.84 | 30.83 | 36.59 | 61.96 | 162.92 | 62.51 |
| Polyfloral | 48.18 | 33.19 | 37.63 | 64.36 | 158.24 | 63.40 |
| Polyfloral | 49.62 | 30.09 | 36.78 | 68.09 | 156.57 | 62.94 |
| Polyfloral | 51.13 | 29.14 | 36.24 | 63.92 | 156.45 | 83.04 |
| Polyfloral | 55.40 | 31.40 | 36.67 | 66.31 | 157.39 | 85.79 |
| Polyfloral | 55.40 | 32.09 | 36.60 | 65.03 | 164.00 | 56.96 |
| Polyfloral | 53.27 | 29.82 | 36.35 | 67.20 | 160.32 | 59.03 |
| Polyfloral | 47.25 | 32.38 | 39.55 | 68.17 | 144.04 | 67.13 |
| Polyfloral | 48.65 | 32.14 | 39.10 | 70.35 | 142.47 | 65.26 |
| Polyfloral | 50.78 | 32.05 | 39.45 | 70.46 | 145.04 | 67.35 |
| Polyfloral | 49.50 | 29.05 | 38.49 | 71.40 | 146.54 | 70.02 |

Appendix Table 3. **Physicochemical properties of *Schefflera abyssinica*** and Polyfloral honey.

| Honey | Refractive index  @ 20 ^0^C | Moisture  (g/100g) | Optical density | pH | Free Acidity  (meq/kg) | Protein  (g/100g) | Ash  (g/100g) | Electrical Conductivity  ( mS/cm) | Diastase Activity (Schade units) | HMF  (mg/Kg) | Color  (Pfund) | Brix  (^0^Brix) |
| --- | --- | --- | --- | --- | --- | --- | --- | --- | --- | --- | --- | --- |
| *Schefflera* | 1.4865 | 20.00 | 0.16 | 3.74 | 32.00 | 0.47 | 0.29 | 0.37 | 3.68 | 11.26 | 52.57 | 78.43 |
| *Schefflera* | 1.487 | 19.80 | 0.18 | 3.72 | 32.00 | 0.49 | 0.28 | 0.35 | 3.82 | 12.76 | 52.23 | 78.61 |
| *Schefflera* | 1.486 | 20.20 | 0.14 | 3.70 | 31.90 | 0.48 | 0.30 | 0.38 | 3.71 | 10.18 | 52.90 | 78.21 |
| *Schefflera* | 1.4855 | 20.40 | 0.14 | 3.73 | 31.60 | 0.49 | 0.32 | 0.39 | 3.80 | 13.55 | 55.56 | 78.06 |
| *Schefflera* | 1.4875 | 19.60 | 0.18 | 3.76 | 32.10 | 0.45 | 0.28 | 0.36 | 3.80 | 10.88 | 54.23 | 78.71 |
| *Schefflera* | 1.4875 | 19.60 | 0.16 | 3.72 | 32.20 | 0.45 | 0.28 | 0.35 | 3.80 | 12.03 | 54.56 | 78.7 |
| *Schefflera* | 1.4865 | 20.00 | 0.15 | 3.78 | 31.90 | 0.48 | 0.32 | 0.39 | 3.68 | 14.81 | 56.22 | 78.44 |
| *Schefflera* | 1.4855 | 20.40 | 0.14 | 3.74 | 31.80 | 0.47 | 0.30 | 0.38 | 3.74 | 11.76 | 54.89 | 78.08 |
| *Schefflera* | 1.487 | 19.80 | 0.16 | 3.82 | 20.00 | 0.45 | 0.24 | 0.29 | 4.42 | 1.99 | 51.57 | 78.6 |
| *Schefflera* | 1.487 | 19.80 | 0.16 | 3.84 | 20.20 | 0.43 | 0.22 | 0.28 | 4.36 | 2.12 | 51.23 | 78.58 |
| *Schefflera* | 1.486 | 20.20 | 0.14 | 3.80 | 20.60 | 0.44 | 0.26 | 0.29 | 4.19 | 2.21 | 51.23 | 78.2 |
| *Schefflera* | 1.4865 | 20.00 | 0.16 | 3.80 | 20.40 | 0.45 | 0.25 | 0.25 | 4.39 | 2.41 | 51.57 | 78.42 |
| *Schefflera* | 1.4875 | 19.60 | 0.18 | 3.86 | 20.10 | 0.42 | 0.27 | 0.26 | 4.42 | 2.73 | 51.90 | 78.7 |
| *Schefflera* | 1.487 | 19.80 | 0.17 | 3.85 | 20.30 | 0.41 | 0.26 | 0.25 | 4.39 | 2.66 | 51.23 | 78.6 |
| *Schefflera* | 1.4865 | 20.00 | 0.16 | 3.79 | 20.00 | 0.43 | 0.24 | 0.27 | 4.22 | 2.15 | 51.57 | 78.44 |
| *Schefflera* | 1.4865 | 20.00 | 0.14 | 3.81 | 20.10 | 0.41 | 0.23 | 0.25 | 4.36 | 2.06 | 50.57 | 78.43 |
| *Schefflera* | 1.4865 | 20.00 | 0.17 | 3.89 | 14.00 | 0.34 | 0.29 | 0.34 | 4.22 | 1.58 | 52.90 | 78.44 |
| *Schefflera* | 1.4855 | 20.40 | 0.15 | 3.90 | 14.10 | 0.33 | 0.30 | 0.35 | 4.30 | 1.65 | 53.56 | 78.04 |
| *Schefflera* | 1.4865 | 20.00 | 0.14 | 3.91 | 14.20 | 0.37 | 0.35 | 0.38 | 4.42 | 1.97 | 56.22 | 78.42 |
| *Schefflera* | 1.4875 | 19.60 | 0.17 | 3.90 | 14.20 | 0.36 | 0.34 | 0.37 | 4.33 | 1.65 | 55.23 | 78.7 |
| Polyfloral | 1.4905 | 18.40 | 0.29 | 3.93 | 28.00 | 0.58 | 0.36 | 0.42 | 5.26 | 3.13 | 129.75 | 80.07 |
| Polyfloral | 1.4895 | 18.80 | 0.26 | 3.95 | 28.20 | 0.57 | 0.37 | 0.44 | 5.18 | 3.30 | 130.41 | 79.57 |
| Polyfloral | 1.4895 | 18.80 | 0.26 | 3.92 | 28.10 | 0.60 | 0.39 | 0.42 | 5.32 | 3.10 | 130.41 | 79.6 |
| Polyfloral | 1.489 | 19.00 | 0.24 | 3.91 | 28.00 | 0.59 | 0.40 | 0.44 | 5.40 | 3.38 | 130.75 | 79.43 |
| Polyfloral | 1.4915 | 18.00 | 0.32 | 3.96 | 28.40 | 0.56 | 0.38 | 0.42 | 5.38 | 4.11 | 130.41 | 80.38 |
| Polyfloral | 1.4905 | 18.40 | 0.30 | 3.96 | 28.60 | 0.56 | 0.37 | 0.41 | 5.43 | 4.03 | 129.75 | 80.06 |
| Polyfloral | 1.491 | 18.20 | 0.29 | 3.93 | 28.50 | 0.59 | 0.36 | 0.40 | 5.46 | 4.67 | 129.42 | 80.22 |
| Polyfloral | 1.4905 | 18.40 | 0.29 | 3.91 | 28.40 | 0.58 | 0.37 | 0.40 | 5.54 | 4.90 | 129.75 | 80.04 |
| Polyfloral | 1.4885 | 19.20 | 0.22 | 4.10 | 20.20 | 0.47 | 0.40 | 0.42 | 5.09 | 6.13 | 130.41 | 79.32 |
| Polyfloral | 1.489 | 19.00 | 0.25 | 4.12 | 20.60 | 0.47 | 0.41 | 0.43 | 5.21 | 6.34 | 131.08 | 79.4 |
| Polyfloral | 1.488 | 19.40 | 0.21 | 4.05 | 20.80 | 0.49 | 0.38 | 0.44 | 4.98 | 6.56 | 130.08 | 79.01 |
| Polyfloral | 1.488 | 19.40 | 0.23 | 4.10 | 20.40 | 0.48 | 0.39 | 0.42 | 5.09 | 6.16 | 130.41 | 79 |
| Polyfloral | 1.4885 | 19.20 | 0.23 | 4.12 | 20.20 | 0.52 | 0.41 | 0.41 | 5.23 | 6.05 | 131.08 | 79.3 |
| Polyfloral | 1.488 | 19.40 | 0.22 | 4.14 | 20.10 | 0.50 | 0.40 | 0.41 | 5.12 | 6.17 | 130.41 | 79.02 |
| Polyfloral | 1.4875 | 19.60 | 0.20 | 4.10 | 20.30 | 0.49 | 0.38 | 0.43 | 5.23 | 6.22 | 130.08 | 78.91 |
| Polyfloral | 1.488 | 19.40 | 0.24 | 4.11 | 20.20 | 0.51 | 0.39 | 0.40 | 5.18 | 6.44 | 130.41 | 79.02 |
| Polyfloral | 1.4895 | 18.80 | 0.28 | 3.98 | 24.20 | 0.39 | 0.51 | 0.46 | 5.01 | 1.84 | 132.41 | 79.58 |
| Polyfloral | 1.4895 | 18.80 | 0.30 | 3.94 | 24.60 | 0.40 | 0.50 | 0.45 | 5.07 | 1.67 | 131.75 | 79.6 |
| Polyfloral | 1.489 | 19.00 | 0.24 | 3.95 | 24.60 | 0.38 | 0.50 | 0.43 | 4.98 | 1.58 | 131.41 | 79.42 |
| Polyfloral | 1.4895 | 18.80 | 0.28 | 3.99 | 24.40 | 0.39 | 0.49 | 0.44 | 5.09 | 1.68 | 131.41 | 79.62 |

Appendix Table 4. **Sugar profile of *Schefflera abyssinica*** and Polyfloral honey.

| Honey | Fructose (%) | Glucose (%) | Sucrose (%) | f:g ratio | Reducing sugar (%) |
| --- | --- | --- | --- | --- | --- |
| *Schefflera* | 38.91 | 31.93 | 0.73 | 1.22 | 71.57 |
| *Schefflera* | 38.70 | 32.09 | 0.76 | 1.21 | 71.55 |
| *Schefflera* | 41.48 | 29.79 | 0.88 | 1.39 | 72.15 |
| *Schefflera* | 42.84 | 29.95 | 0.94 | 1.43 | 73.73 |
| *Schefflera* | 41.83 | 30.36 | 0.74 | 1.38 | 72.93 |
| *Schefflera* | 42.91 | 30.44 | 0.78 | 1.41 | 74.13 |
| *Schefflera* | 42.63 | 30.76 | 0.72 | 1.39 | 74.11 |
| *Schefflera* | 41.85 | 30.82 | 0.64 | 1.36 | 73.31 |
| *Schefflera* | 38.28 | 28.39 | 0.70 | 1.35 | 67.37 |
| *Schefflera* | 38.42 | 28.03 | 0.74 | 1.37 | 67.19 |
| *Schefflera* | 39.01 | 28.21 | 0.74 | 1.38 | 67.96 |
| *Schefflera* | 39.17 | 28.27 | 0.70 | 1.39 | 68.14 |
| *Schefflera* | 39.47 | 28.26 | 0.68 | 1.40 | 68.41 |
| *Schefflera* | 39.55 | 27.84 | 0.70 | 1.42 | 68.09 |
| *Schefflera* | 39.05 | 27.68 | 0.58 | 1.41 | 67.31 |
| *Schefflera* | 38.97 | 28.06 | 0.56 | 1.39 | 67.59 |
| *Schefflera* | 38.52 | 29.14 | 0.38 | 1.32 | 68.04 |
| *Schefflera* | 38.66 | 29.04 | 0.32 | 1.33 | 68.02 |
| *Schefflera* | 38.89 | 29.08 | 0.34 | 1.34 | 68.31 |
| *Schefflera* | 38.58 | 29.45 | 0.38 | 1.31 | 68.41 |
| Polyfloral | 34.89 | 32.76 | 0.28 | 1.06 | 67.93 |
| Polyfloral | 35.27 | 33.06 | 0.26 | 1.07 | 68.59 |
| Polyfloral | 35.77 | 33.35 | 0.34 | 1.07 | 69.46 |
| Polyfloral | 36.16 | 33.31 | 0.32 | 1.09 | 69.79 |
| Polyfloral | 36.35 | 33.63 | 0.38 | 1.08 | 70.36 |
| Polyfloral | 36.77 | 33.87 | 0.38 | 1.09 | 71.02 |
| Polyfloral | 36.51 | 33.63 | 0.36 | 1.09 | 70.50 |
| Polyfloral | 36.83 | 34.05 | 0.34 | 1.08 | 71.22 |
| Polyfloral | 36.41 | 34.27 | 0.30 | 1.06 | 70.98 |
| Polyfloral | 36.53 | 34.57 | 0.32 | 1.06 | 71.42 |
| Polyfloral | 36.88 | 34.49 | 0.26 | 1.07 | 71.63 |
| Polyfloral | 36.82 | 34.75 | 0.26 | 1.06 | 71.83 |
| Polyfloral | 37.01 | 33.53 | 0.30 | 1.10 | 70.84 |
| Polyfloral | 36.57 | 33.97 | 0.34 | 1.08 | 70.88 |
| Polyfloral | 36.51 | 33.91 | 0.36 | 1.08 | 70.78 |
| Polyfloral | 36.55 | 34.66 | 0.36 | 1.05 | 71.57 |
| Polyfloral | 36.43 | 34.87 | 0.34 | 1.04 | 71.64 |
| Polyfloral | 36.17 | 34.91 | 0.32 | 1.04 | 71.40 |
| Polyfloral | 36.00 | 33.74 | 0.38 | 1.07 | 70.12 |
| Polyfloral | 36.15 | 33.38 | 0.36 | 1.08 | 69.89 |

Appendix **Figures**


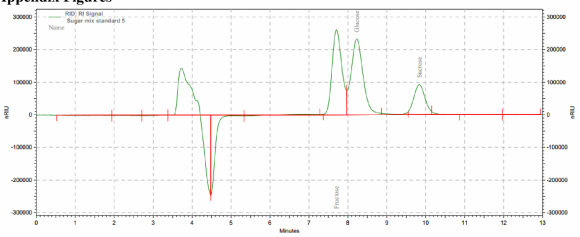


Appendix Figure 1. HPLC Chromatogram of Sugar standard (where retention time of Fructose = 7.465, Glucose = 8.106 and Sucrose = 9. 527).


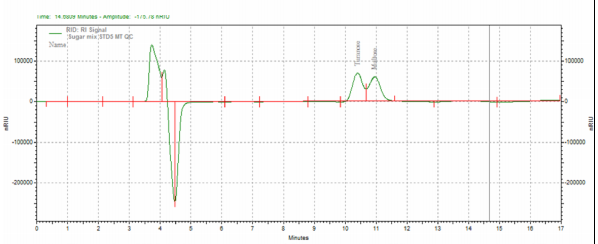


Appendix Figure 2. HPLC Chromatogram of Sugar standard (Where retention time of Turanose = 10. 103 and Maltose = 10. 625).


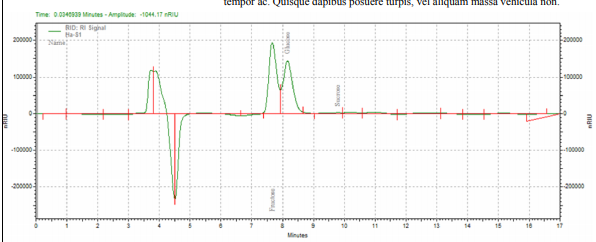


Appendix Figure 3. HPLC Chromatogram of Sugar in *Schefflera abyssinica* honey (where retention time of Fructose = 7.465, Glucose = 8.106 and Sucrose = 9. 527).


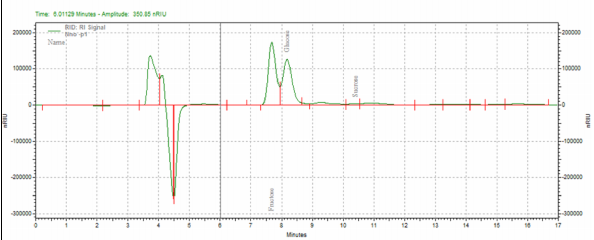


Appendix Figure 4. HPLC Chromatogram of Sugar in polyfloral honey (where retention time of Fructose = 7.465, Glucose = 8.106 and Sucrose = 9. 527).


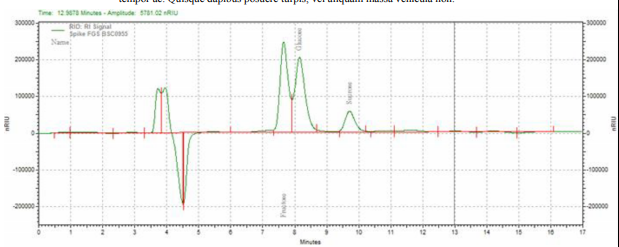


Appendix Figure 5. HPLC Chromatogram of Sugars in spiked honey sample for quality control (where retention time of Fructose = 7.465, Glucose = 8.106 and Sucrose = 9. 527).


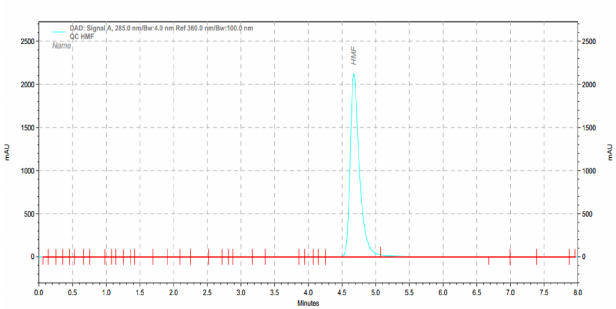


Appendix Figure 6. HPLC Chromatogram of HMF standard at retention time of 4. 611.


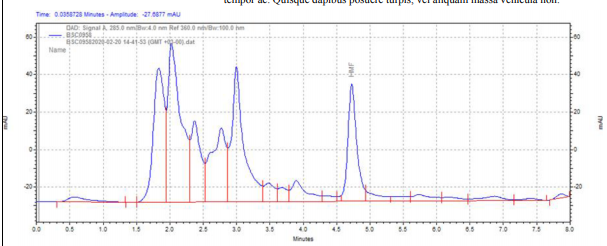


Appendix Figure 7. HPLC Chromatogram of HMF for *Schefflera abyssinica* honey (where HMF at retention time of 4. 611).


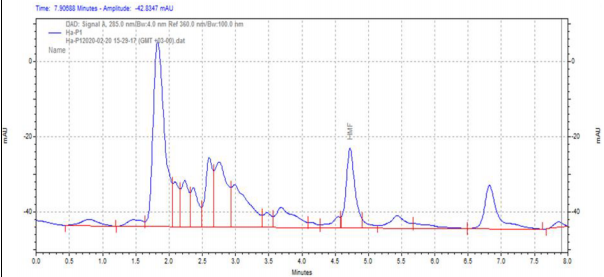


Appendix Figure 8. HPLC Chromatogram of HMF for Polyfloral honey (where HMF at retention time of 4. 611).


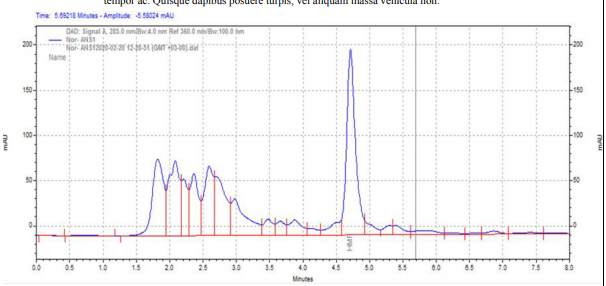


Appendix Figure 9. HPLC Chromatogram of HMF in Spiked honey sample for quality control (where HMF at retention time of 4. 611).


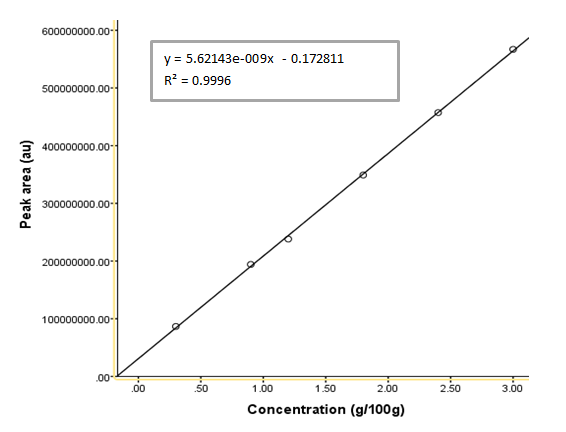
Appendix Figure 10. Calibration graph of fructose in g/100g


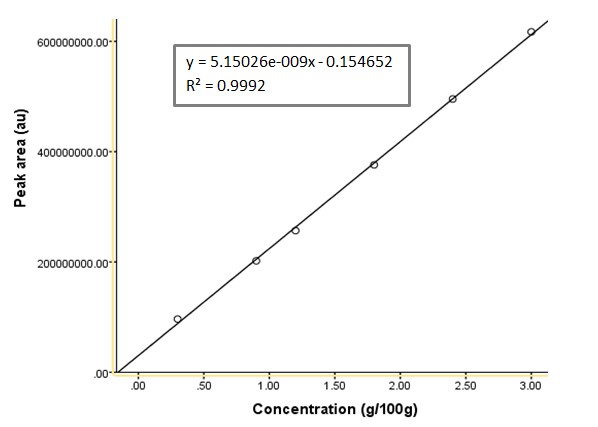


Appendix Figure 11. Calibration graph of glucose in g/100g


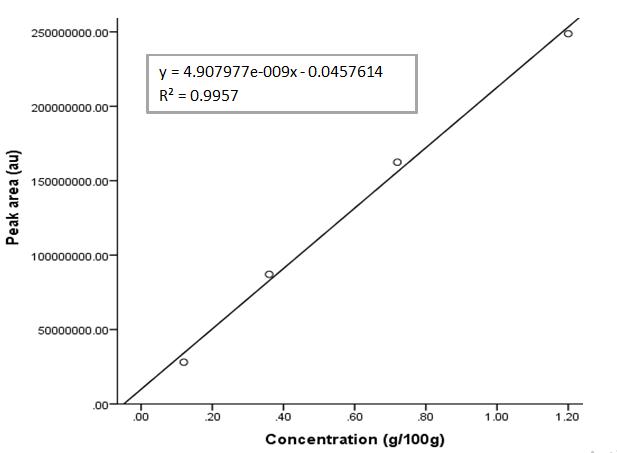


Appendix Figure 12. Calibration graph of sucrose in g/100g


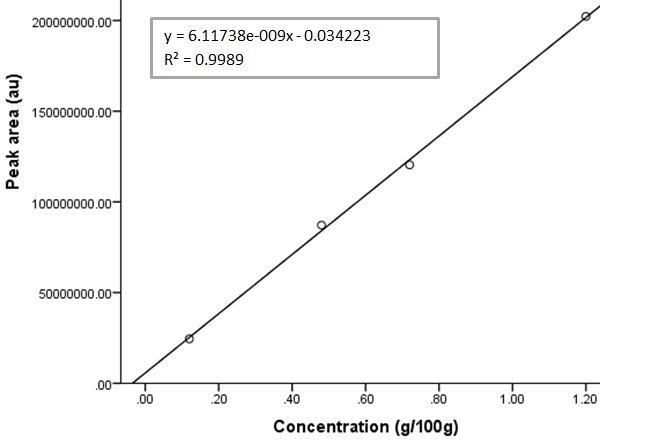


Appendix Figure 13. Calibration graph of turanose in g/100g


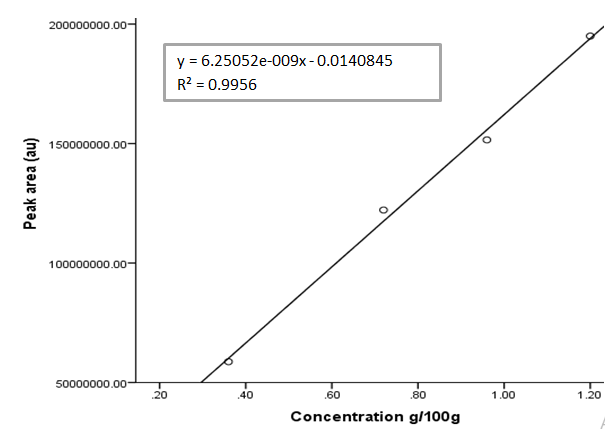


Appendix Figure 14. Calibration graph of maltose in g/100g


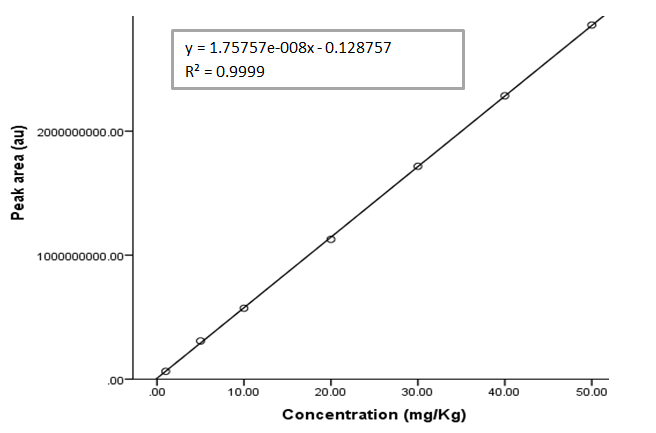


Appendix Figure 15. Calibration graph of HMF in mg/Kg

ANOVA table for Refractive index

| Source | DF | Sum of Squares | Mean Square | F Value | Pr > F |
| --- | --- | --- | --- | --- | --- |
| Model | 1 | 0.00 | 0.00 | 83.26 | <.0001 |
| Error | 38 | 0.00 | 0.00 |  |  |
| Total | 39 | 0.00 |  |  |  |

ANOVA table for moisture

| Source | DF | Sum of Squares | Mean Square | F Value | Pr > F |
| --- | --- | --- | --- | --- | --- |
| Model | 1 | 11.24 | 11.24 | 83.26 | <.0001 |
| Error | 38 | 5.13 | 0.13 |  |  |
| Total | 39 | 16.36 |  |  |  |

ANOVA table for optical density

| Source | DF | Sum of Squares | Mean Square | F Value | Pr > F |
| --- | --- | --- | --- | --- | --- |
| Model | 1 | 0.10 | 0.10 | 143.13 | <.0001 |
| Error | 38 | 0.03 | 0.00 |  |  |
| Total | 39 | 0.13 |  |  |  |

ANOVA table for pH

| Source | DF | Sum of Squares | Mean Square | F Value | Pr > F |
| --- | --- | --- | --- | --- | --- |
| Model | 1 | 0.42 | 0.42 | 72.46 | <.0001 |
| Error | 38 | 0.22 | 0.01 |  |  |
| Total | 39 | 0.64 |  |  |  |

ANOVA table for free acidity

| Source | DF | Sum of Squares | Mean Square | F Value | Pr > F |
| --- | --- | --- | --- | --- | --- |
| Model | 1 | 4.29 | 4.29 | 0.13 | 0.7210 |
| Error | 38 | 1259.61 | 33.15 |  |  |
| Total | 39 | 1263.90 |  |  |  |

ANOVA table for crude protein

| Source | DF | Sum of Squares | Mean Square | F Value | Pr > F |
| --- | --- | --- | --- | --- | --- |
| Model | 1 | 0.06 | 0.06 | 14.59 | 0.0005 |
| Error | 38 | 0.15 | 0.00 |  |  |
| Total | 39 | 0.21 |  |  |  |

ANOVA table for crude ash

| Source | DF | Sum of Squares | Mean Square | F Value | Pr > F |
| --- | --- | --- | --- | --- | --- |
| Model | 1 | 0.16 | 0.16 | 86.69 | <.0001 |
| Error | 38 | 0.07 | 0.00 |  |  |
| Total | 39 | 0.23 |  |  |  |

ANOVA table for Electrical conductivity

| Source | DF | Sum of Squares | Mean Square | F Value | Pr > F |
| --- | --- | --- | --- | --- | --- |
| Model | 1 | 0.09 | 0.09 | 60.73 | <.0001 |
| Error | 38 | 0.06 | 0.00 |  |  |
| Total | 39 | 0.15 |  |  |  |

ANOVA table for Diastase activity

| Source | DF | Sum of Squares | Mean Square | F Value | Pr > F |
| --- | --- | --- | --- | --- | --- |
| Model | 1 | 12.32 | 12.32 | 208.85 | <.0001 |
| Error | 38 | 2.24 | 0.06 |  |  |
| Total | 39 | 14.56 |  |  |  |

ANOVA table for HMF

| Source | DF | Sum of Squares | Mean Square | F Value | Pr > F |
| --- | --- | --- | --- | --- | --- |
| Model | 1 | 30.54 | 30.54 | 2.05 | 0.1604 |
| Error | 38 | 566.01 | 14.90 |  |  |
| Total | 39 | 596.55 |  |  |  |

ANOVA table for fructose

| Source | DF | Sum of Squares | Mean Square | F Value | Pr > F |
| --- | --- | --- | --- | --- | --- |
| Model | 1 | 126.52 | 126.52 | 84.03 | <.0001 |
| Error | 38 | 57.22 | 1.50 |  |  |
| Total | 39 | 183.74 |  |  |  |

ANOVA table for glucose

| Source | DF | Sum of Squares | Mean Square | F Value | Pr > F |
| --- | --- | --- | --- | --- | --- |
| Model | 1 | 207.57 | 207.57 | 188.86 | <.0001 |
| Error | 38 | 41.76 | 1.10 |  |  |
| Total | 39 | 249.33 |  |  |  |

ANOVA table for sucrose content

| Source | DF | Sum of Squares | Mean Square | F Value | Pr > F |
| --- | --- | --- | --- | --- | --- |
| Model | 1 | 1.04 | 1.04 | 65.37 | <.0001 |
| Error | 38 | 0.60 | 0.01 |  |  |
| Total | 39 | 1.64 |  |  |  |

ANOVA table for fructose: glucose ratio

| Source | DF | Sum of Squares | Mean Square | F Value | Pr > F |
| --- | --- | --- | --- | --- | --- |
| Model | 1 | 0.83 | 0.83 | 438.49 | <.0001 |
| Error | 38 | 0.07 | 0.00 |  |  |
| Total | 39 | 0.90 |  |  |  |

ANOVA table for phenol

| Source | DF | Sum of Squares | Mean Square | F Value | Pr > F |
| --- | --- | --- | --- | --- | --- |
| Model | 1 | 5967.76 | 5967.76 | 1029.89 | <.0001 |
| Error | 38 | 220.19 | 5.79 |  |  |
| Total | 39 | 6187.95 |  |  |  |

ANOVA table for flavonoid

| Source | DF | Sum of Squares | Mean Square | F Value | Pr > F |
| --- | --- | --- | --- | --- | --- |
| Model | 1 | 1201.32 | 1201.32 | 611.83 | <.0001 |
| Error | 38 | 74.61 | 1.96 |  |  |
| Total | 39 | 1275.94 |  |  |  |

ANOVA table for DPPH

| Source | DF | Sum of Squares | Mean Square | F Value | Pr > F |
| --- | --- | --- | --- | --- | --- |
| Model | 1 | 422.24 | 422.24 | 376.17 | <.0001 |
| Error | 38 | 42.65 | 1.12 |  |  |
| Total | 39 | 464.89 |  |  |  |

ANOVA table for H_2_O_2_

| Source | DF | Sum of Squares | Mean Square | F Value | Pr > F |
| --- | --- | --- | --- | --- | --- |
| Model | 1 | 1160.90 | 1160.90 | 73.00 | <.0001 |
| Error | 38 | 604.27 | 15.90 |  |  |
| Total | 39 | 1765.17 |  |  |  |
